# Birth prevalence and determinants of neural tube defects among newborns in Ethiopia: A systematic review and meta-analysis

**DOI:** 10.1371/journal.pone.0315122

**Published:** 2025-01-02

**Authors:** Beminet Moges Gebremariam, Dejene Hailu, Barbara J. Stoecker, Afework Mulugeta

**Affiliations:** 1 School of Public Health, College of Medicine and Health Sciences, Wachemo University, Hossana, Ethiopia; 2 School of Public Health, College of Medicine and Health Sciences, Hawassa University, Hawassa, Ethiopia; 3 Department of Nutritional Sciences, Oklahoma State University, Stillwater, Oklahoma, United States of America; 4 Department of Public Health Sciences, College of Medicine and Health Sciences, Mekelle University, Mekelle, Ethiopia; Bahir Dar University, ETHIOPIA

## Abstract

**Background:**

Neural tube defects (NTDs) are complex multifactorial disorders in the neurulation of the brain and spinal cord that develop in humans between 21 and 28 days of conception. Neonates with NTDs may experience morbidity and mortality, with severe social and economic consequences. Therefore, the aim of this systematic review and meta-analysis is to assess the pooled prevalence and determinants for neural tube defects among newborns in Ethiopia.

**Methods:**

The protocol of this study was registered in the International Prospective Register of Systematic Reviews (PROSPERO Number: CRD42023407095). We systematically searched the databases PubMed, Science Direct, Cochrane Library, Google Scholar and Research Gate. Grey literature was searched on Google. Heterogeneity among studies was assessed using the I^2^ test statistic and the Cochran Q test statistic. A random effects model was used to estimate the birth prevalence of neural tube defects.

**Result:**

Twenty-five articles were included in the meta-analysis to estimate the prevalence and determinants of neural tube defects in Ethiopia. A total of 611,354 newborns were included in the analysis. The pooled birth prevalence of neural tube defects was 83.40 (95% CI: 60.78, 106.02) per 10,000 births. The highest and lowest prevalence rates were 130.9 (95% CI: 113.52, 148.29) in Tigray and 28.60 (95% CI: 18.70, 38.50) per 10,000 births in Amhara regional states. Women’s intake of folic acid supplements and planned pregnancy were identified as protective factors for NTDs, while stillbirth history, use of any drugs during pregnancy, exposure to radiation, and pesticides during pregnancy were risk factors for neural tube defects.

**Conclusion:**

The pooled birth prevalence of neural tube defects in Ethiopia was found to be high. Effective prevention interventions, especially focusing on periconceptional folic acid supplementation as well as folate fortification, should be prioritized alongside nutrition education, maternal health care, and environmental safety measures.

## Background

Neural tube defects (NTDs) are complex multifactorial disorders in neurulation of the brain and spinal cord that develop between 21 and 28 days after conception in humans [1]. Neural tube defects are among the most serious and prevalent types of congenital disorders [2]. Neural tube defects are categorized into two major groups. Anencephaly and encephalocele are brain structure anomalies, while meningocele, myelomeningocele, and other forms of spina bifida are spinal cord structure defects [3, 4].

Globally, an estimated 300,000 babies are born with neural tube defects each year, which equates to 8.6 million disability-adjusted life years [5]. In Africa, NTDs are the most common birth defects, affecting approximately 1–3 per 1000 births each year [6]. In low-income countries, NTDs may account for 29% of neonatal deaths due to observable birth defects [7].

Children with NTDs typically have little to no bladder and/or bowel control, abnormalities of the hips, knees, and feet, and anesthesia of the skin [8]. Newborns with anencephaly die soon after delivery, while those with spina bifida have the greatest social and economic impact, typically connected with professional stress, loss of human potential, and expensive medical care expenditures [9]. The lifetime direct medical cost for patients with NTDs is high, with the majority of costs spent during inpatient care, childhood therapy, and adult comorbidity management [10].

Multiple factors are involved in the causation of NTDs. The predominant factors include genetics and environment [11, 12]. Genetic components for NTDs are demonstrated by a 2–5% risk of recurrence in siblings and a family history in many affected cases [13]. NTDs have been linked to trisomies 13 and 18 genetic disorders, as well as chromosomal rearrangements [14]. As a result of folate’s crucial function in methylation metabolism, environmental factors including maternal folate status; are linked to the risk of NTDs in addition to genetic factors [15, 16]. Other maternal factors, including chronic diseases such as diabetes and obesity before pregnancy, have been related to the etiology of NTDs [17, 18]. Additionally, certain anti-epileptic medications contribute to the burden of NTDs, and may interfere with folate metabolism by inhibiting dihydrofolate reductase (DHFR) [19, 20]. Furthermore, pesticide exposure in the mother, whether occupational or through food/water consumption, is a teratogen associated with increased risk of NTDs [21–23].

A daily dose of 400 μg of folic acid can prevent up to 72% of neural tube defects [24, 25]. Periconceptional folic acid supplementation led to significant decreases in the rates of anencephaly [26]. Evidence indicates that most neural tube defects are preventable if women consume enough folic acid supplements prior to and during the first 3 months of pregnancy [27].

A few studies in Ethiopia found that the Oromia region had the highest frequency of NTDs, with 167.4 cases per 10,000 newborns, followed by the Tigray region, which had 130.8 cases per 10,000 births [28, 29]. According to a nationwide study in Ethiopia, a large number of women are folate deficient and at an increased risk of NTDs [30]. Despite global recommendations, folic acid supplementation coverage and adherence remain low [31–33]. In addition, Ethiopia has failed to prioritize periconceptional folic acid supplementation and remains one of the countries without mandatory folate fortification. Moreover, there is a dearth of aggregated data on the burden of NTDs and the various risk factors associated with their prevalence throughout the country. As a result, the aim of this systematic review and meta-analysis is to assess the pooled prevalence and determinants for neural tube defects in newborns in Ethiopia.

## Methods

### Study protocol

This systematic review and meta-analysis were conducted using the guidelines of Preferred Reporting Items for Systematic Reviews and Meta-Analysis (PRISMA) [34] (S1 Table). The protocol for this study was registered in the International Prospective Register of Systematic Reviews (PROSPERO), with the University of York Centre for Reviews and Dissemination (ID Number: CRD42023407095) [35].

### Review of outcomes

The primary goal of the review was to determine the pooled prevalence of neural tube abnormalities. We presented an overview of the overall and subtype prevalence of neural tube defects in newborns in Ethiopia. The main outcome was established as the pooled proportion of neural tube defects per 10,000 births. The secondary outcome was a pooled estimate of the relation between neural tube defects and selected risk factors in Ethiopia.

### Search strategy

A systematic review and meta-analysis was conducted using published and unpublished articles on the prevalence of neural tube defects and determinants in Ethiopia. The databases used to search for studies were PubMed, Science Direct, Cochrane Library, Google Scholar and Research Gate. Grey literature was searched on Google. The Boolean operators "AND" and "OR" were used alone or in combination with the following key terms: "birth prevalence" OR "burden" AND "neural tube defect" AND "associated factors" OR "determinants" OR "risk factors" AND "newborns" OR "neonates" AND "Ethiopia" OR "Regional states in Ethiopia" were primarily used for PubMed. The other databases’ article searches employed interchangeable main search phrases (prevalence, neural tube defects, related factors/determinants/risk factors, infants, and Ethiopia) (S1 Appendix). Afterwards, all identified studies from the databases were listed alongside the reasons for exclusion (S2 Appendix). The relevant articles were systematically searched across all specified databases from April 14 to August 24, 2023.

### Eligibility criteria

Any research conducted in Ethiopia that reported the prevalence and determinants of neural tube defects that fulfilled the selection criteria was examined for analysis.

### Study area, designs, and population

In this systematic review and meta-analysis, we included all studies conducted in Ethiopia. All descriptive and observational types of epidemiological studies (cross-sectional, case-control, and cohort) reporting the prevalence and determinants of neural tube defects were eligible for this systematic review and meta-analysis.

### Language and publication status

Only English-language articles were considered. The review includes articles that were both published and unpublished.

### Study period

In this review, there was no restriction on the study period.

### Study selection procedure

In this study, cross-sectional, cohort, and case-control studies were included in the final analysis. The studies were reviewed using the PICOS (participants, interventions, comparison, outcomes, and study setting) criteria. Studies that reported the prevalence or magnitude of neural tube defects and associated factors that were done in different regions of Ethiopia were selected. To begin, articles extracted from different sources were exported to the EndNoteX8 citation manager. Then, the duplicates were removed. The titles and abstracts of the studies then were screened with predefined inclusion criteria. Lastly, two authors (BM and DH) reviewed the full texts of the selected studies.

### Risk of bias assessment

The risk of bias was evaluated using the Joanna Briggs Institute (JBI) critical evaluation tool [36]. The authors (BM and DH) assessed the quality of the complete text considered for inclusion in the meta-analysis. The instrument includes ten items for case-control, eight for cross-sectional, and eleven for cohort research. Each item was rated as yes (1) or no (0). When the information presented was insufficient to make a decision about a single issue, the grade for that item was ’No’ (0). Each study was assessed based on the number of items scored ’yes’ (1). Cross-sectional studies were categorized as low (≥7), medium (5–6), or high (≤4). Case-control studies were grouped into three categories: low (≥8), medium (7–6), and high (≤5). Similarly, we classified the cohort studies as low risk (≥8), medium risk (7–6), or high risk (≤5) (S2 Table).

### Data extraction

The selected research articles were rigorously evaluated, and the data needed for the systematic review was summarized in table format using Microsoft Office Excel. Data extraction was performed by two independent reviewers (BMG and DH) between August 28 and September 16, 2023, using a standardized data extraction tool developed by JBI. Data extracted included the author(s)’ names, publication year, study design, sample size, NTD prevalence, factors, and odds ratio (ORs) (S3 Table). Thirteen (52%) investigations was conducted between 2015 and 2019, with 12 (48%) studies taking place between 2020 and 2022.

### Statistical methods and analysis

The final extracted data was entered into STATA version 17 software for statistical analysis. The I^2^ and Cochran Q test statistics were used to measure heterogeneity among studies, while Begg’s test statistics were used to detect publication bias [37–39]. I^2^ scores of 25%, 50%, and 75% were considered as indicating low, medium, or high heterogeneity. This meta-analysis validated and justified heterogeneity for I^2^ > 50% and P value < 0.05. [40]. Pooled odds ratios (ORs) with 95% confidence intervals (CIs) were used to assess the relationship between determinant factors and neural tube defects. Subgroup analysis for NTD prevalence were conducted by year of publication and Ethiopian region. We ran a sensitivity analysis to assess the study’s impact on the total pooled estimates, and the results are shown graphically.

### Operational definition

Birth prevalence of neural tube defects is the number of NTD cases (both live births and stillbirths) per 10,000 total births during the same year.

## Results

### Characteristics of the included studies

**Fig 1:**
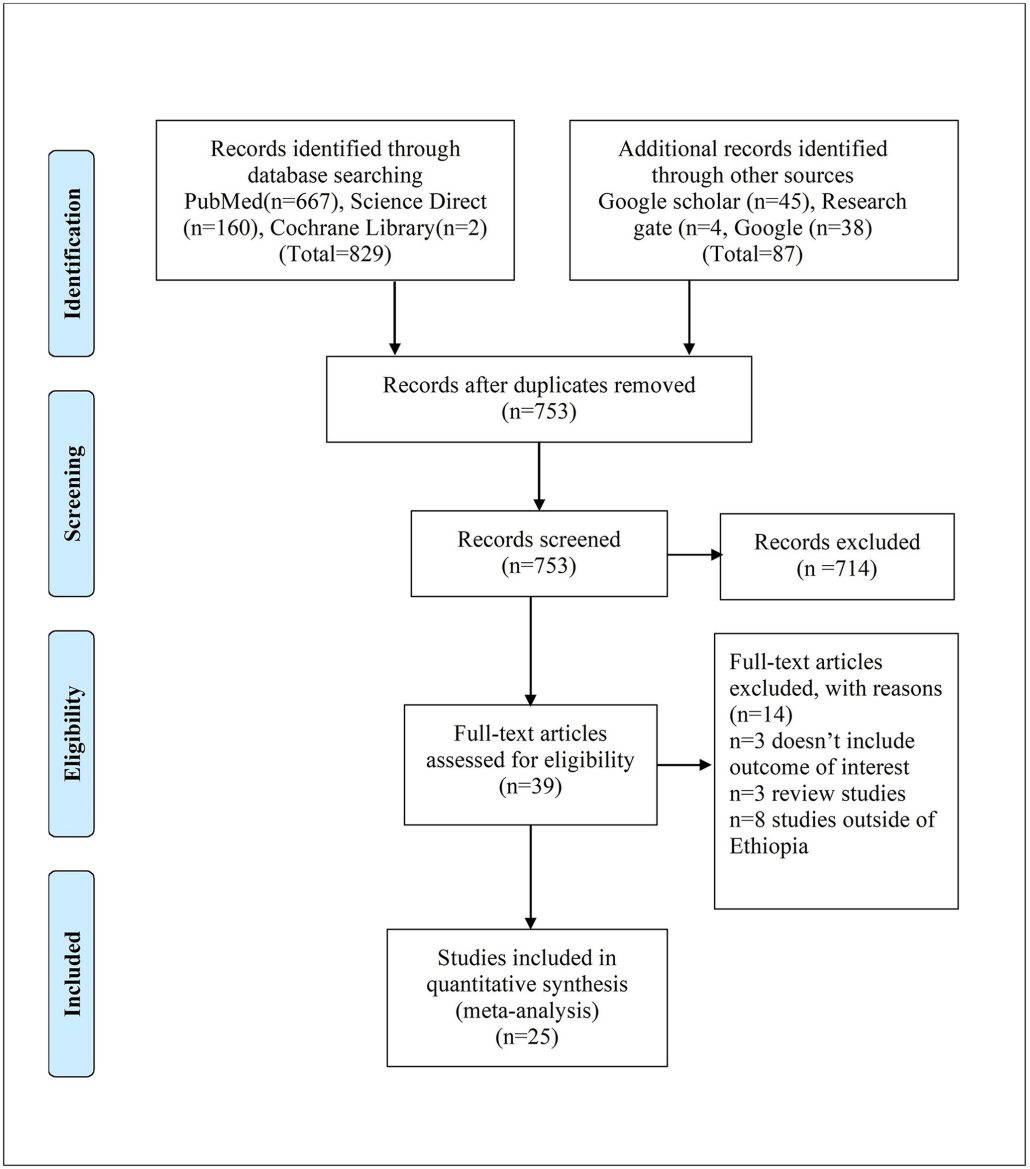
PRISMA flow diagram for birth prevalence of neural tube defects in Ethiopia 2023.

A total of 916 studies were obtained in the searching of databases and other sources, and 163 duplicates were removed. After screening using titles and abstracts, 39 studies were checked for eligibility through a full text assessment (Fig 1).

Finally, twenty-five studies were included in the final analysis. Fourteen were cross-sectional, 2 were cohort studies and nine were case-control studies. All of the studies were conducted in health facilities, and the study populations were newborns and children aged 0–17 years. In the final analysis of the systematic review and meta-analysis, fifteen of the studies in the analysis of pooled birth prevalence of NTDs were conducted in Addis Ababa, Amhara, Oromia, and Tigray regions of Ethiopia [28, 29, 41–53]. Determinants of NTDs were reported in 9 case-control studies [22, 23, 45, 53, 55, 57–60] and 1 cross-sectional study [56], done in 4 different regions of the country (Tables 1 and 2).

**Table 1:** Characteristics of included studies for prevalence of neural tube defects in Ethiopia, 2023.

Authors	Year	Region	Design	population	Sample size	NTDs Prevalence per 10,000	Anencephaly per 10,000	Spinal bifida per 10,000	Encephalocele per10,000	Risk of bias
Sorri et al. [41]	2015	Addis Ababa	Cross-sectional	newborn	28961	61.1	32.8	28.3	1.7	Medium
Mekonen et al. [42]	2015	Tigray	Prospective cohort	newborn	1516	131.9	6.6	125.3	-	High
Taye et al. [43]	2016	Addis Ababa and Amhara	Cross-sectional	children 0–17 years	319776	31.6	5.7	31.1	0.8	Medium
Mitiku et al. [44]	2017	Addis Ababa	Cross-sectional	newborn	84	238.1	238.1	-	-	High
Berihu et al. [29]	2018	Tigray	Cross-sectional	newborn	14903	130.8	66.4	64.4	-	Medium
Gedefaw et al. [45]	2018	Addis Ababa	Cross-sectional	newborn	8677	127.9	69.1	51.9	6.9	Medium
Adane et al. [46]	2018	Amhara	Cross-sectional	newborn	19650	52.4	5.1	30.5	-	Medium
Legese et al. [47]	2019	Addis Ababa	Cross-sectional	newborn	876	68.5	34.2	11.4	11.4	Medium
Taye et al. [48]	2019	Addis Ababa and Amhara	Cross-sectional	children 0–17 years	76201	80.3	4.7	35.2	-	Low
Abdu et al. [49]	2019	Amhara	Cross-sectional	newborn	22624	53.5	-	53.5	-	Medium
Genti et al. [50]	2021	Oromia	Cross-sectional	newborn	45951	40.5	13.7	11.1	-	Medium
Silesh et al. [28]	2021	Oromia	Cross-sectional	newborn	3346	167.4	26.9	86.7	-	Medium
Mekonnen et al. [51]	2021	Amhara	Cross-sectional	newborn	11177	28.6	15.2	13.4	-	Low
Kindie et al. [52]	2022	Amhara	Cross-sectional	newborn	8862	109.5	56.4	34.9	10.2	Medium
Berhane et al. [53]	2022	Oromia	Retrospective cohort	newborn	48750	107.5	51.7	40.8	6.2	Medium

**Table 2:** Characteristics of included studies for determinants of neural tube defects in Ethiopia, 2023.

Authors	Design	Study Area	Population	Total samples	Study Population		Factors	OR (95%CI)	Risk of bias
					Case	Control			
Berihu et al. [54]	Case-control	Tigray	Newborn-mothers	617	205	412	Maternal age < 35 yrs	2.46 (1.33,4.53)	Medium
							Folic acid tablets	2.15 (1.02,4.54)	
							Previous history of stillbirth	19.1 (4.28,85.48)	
							Exposure to radiation	5 (0.150,166.60)	
							Maternal alcohol consumption	10.3(1.19,88.50)	
							Exposure to pesticide	5 (0.150,166.60)	
							Unplanned pregnancy	1.97 (1.38,2.82)	
Gedefaw et.al. [45]	Case-control	Addis Ababa	Newborn-mothers	333	111	222	Maternal age < 35yrs	2(0.73,5.47)	Low
							Folic acid tablets	0.47 (0.23,0.95)	
							Previous history of stillbirth	0.49(0.10,2.35)	
							Planned pregnancy	0.47 (0.24,0.92)	
Atlaw et al. [22]	Case-control	Oromia	Newborn-mothers	462	42	420	Maternal age |<35 yrs	4.77 (1.10–20.66)	Low
							Folic acid tablets	0.09 (.031, 285)	
							Previous history of stillbirth	1.41 (.42,4.75)	
							Exposure to radiation	0.44 (.09, 2.08)	
							Alcohol consumption	0.79 (.32,1.98)	
							Exposure to pesticide	0.19 (.02,2.2)	
Tadesse et al. [55]	Case-control	Amhara	Newborn-mothers	400	133	267	Rural residence	1.78(1.02,3.11)	Low
							Folic acid tablets	0.37(0.21,0.65)	
							Unplanned pregnancy	0.94 (0.54,1.66)	
							Never took any substance	0.42 (0.21,0.88)	
Edris et al. [56]	Cross-sectional	Oromia	Newborn-mothers	420	-	-	Maternal age< 35 yrs	3.84 (2.1,10.7)	Low
							Urban residence	0.48 (0.2,3.7)	
							Radiation exposure	5.01 (1.6,14.3)	
							AEDs drug intake	4.75 (1.5,16.2)	
Abebe et al. [57]	Case-control	Amhara	Newborn-mothers	123	41	82	Maternal age less than 35 years old	0.13(0.02,0.72)	Low
							Any drug intake	3.73(1.48,9.41)	
Tesfaye et al. [58]	Case-control	Addis Ababa	Newborn-mothers	180	60	120	Maternal age < 35yrs	33.7 (2.53,448.5)	Low
							Planned pregnancy	0.41 (0.08,2.30)	
							Passive cigarette smoking	1.0(0.23,9.91)	
Gashaw et al. [23]	Case-control	Amhara	Newborn-mothers	243	81	162	Family annual income < 24000ETB	3.73(1.35,10.26)	Low
							History of still birth	3.63(1.03,12.2)	
							History of abortion	6.15(2.63,18.56)	
							Pesticides exposure	5.34(1.77,16.05)	
							preconception care	0.14(0.05,0.39)	
							Folic acid tablets	0.16 (0.07,0.33)	
Getinet et al. [59]	Case-control	Oromia	Newborn-mothers	219	74	145	Rural residence	7.99 (3.99,16.03)	Low
Mulu et al. [60]	Case-control	Amhara	Newborn-mothers	381	127	254	Intake of medication during pregnancy	1.83(1.08,3.08)	Low
							Mothers who did not take a balanced diet	13.46(7.83,23.13)	
							Folic acid supplementation	1.71 (1.01,2.94)	

### Pooled prevalence of neural tube defects (NTDs) in Ethiopia

**Fig 2:**
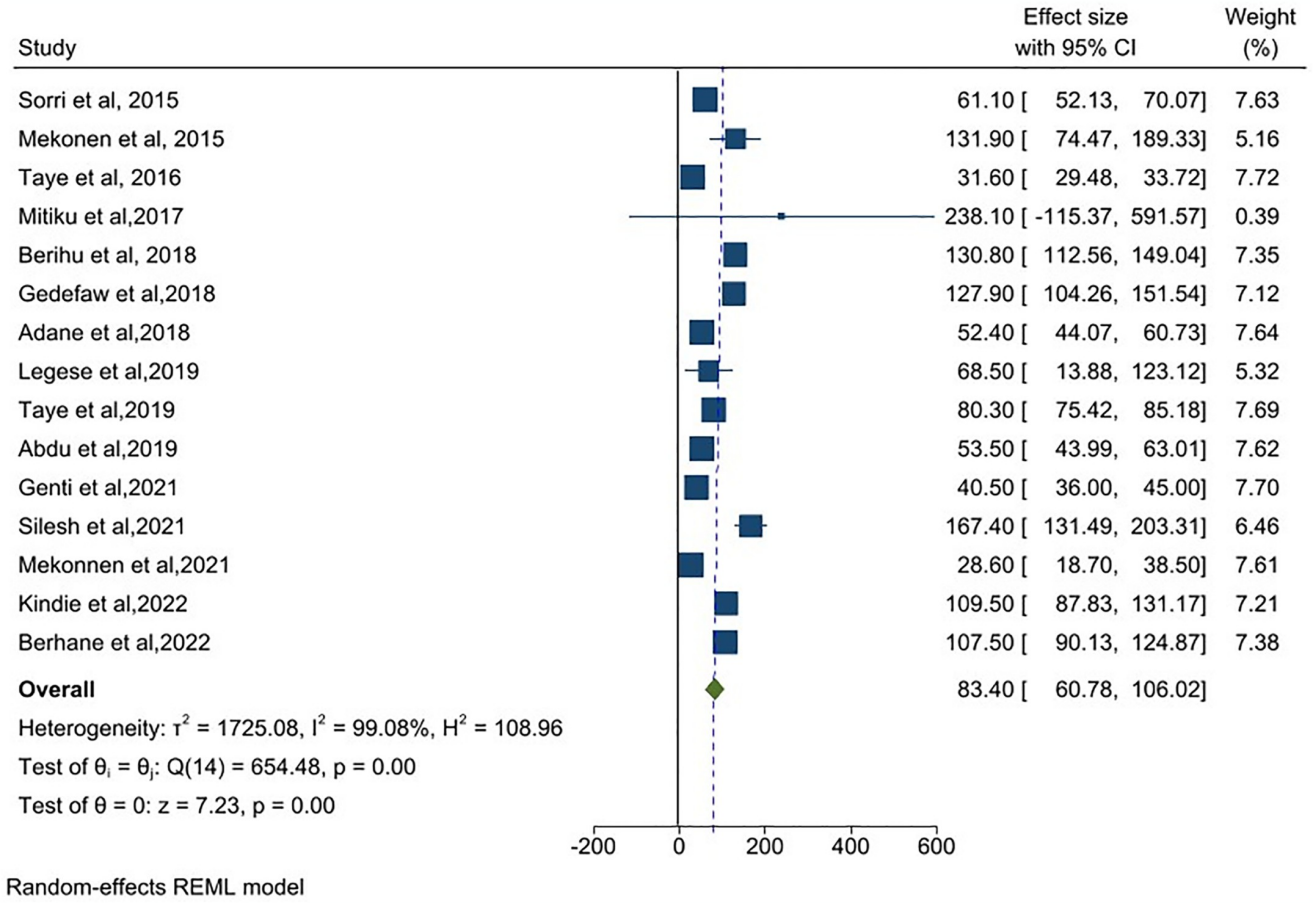
Meta-analysis, birth prevalence of Neural tube defect per 10,000 births in Ethiopia, 2023.

**Fig 3:**
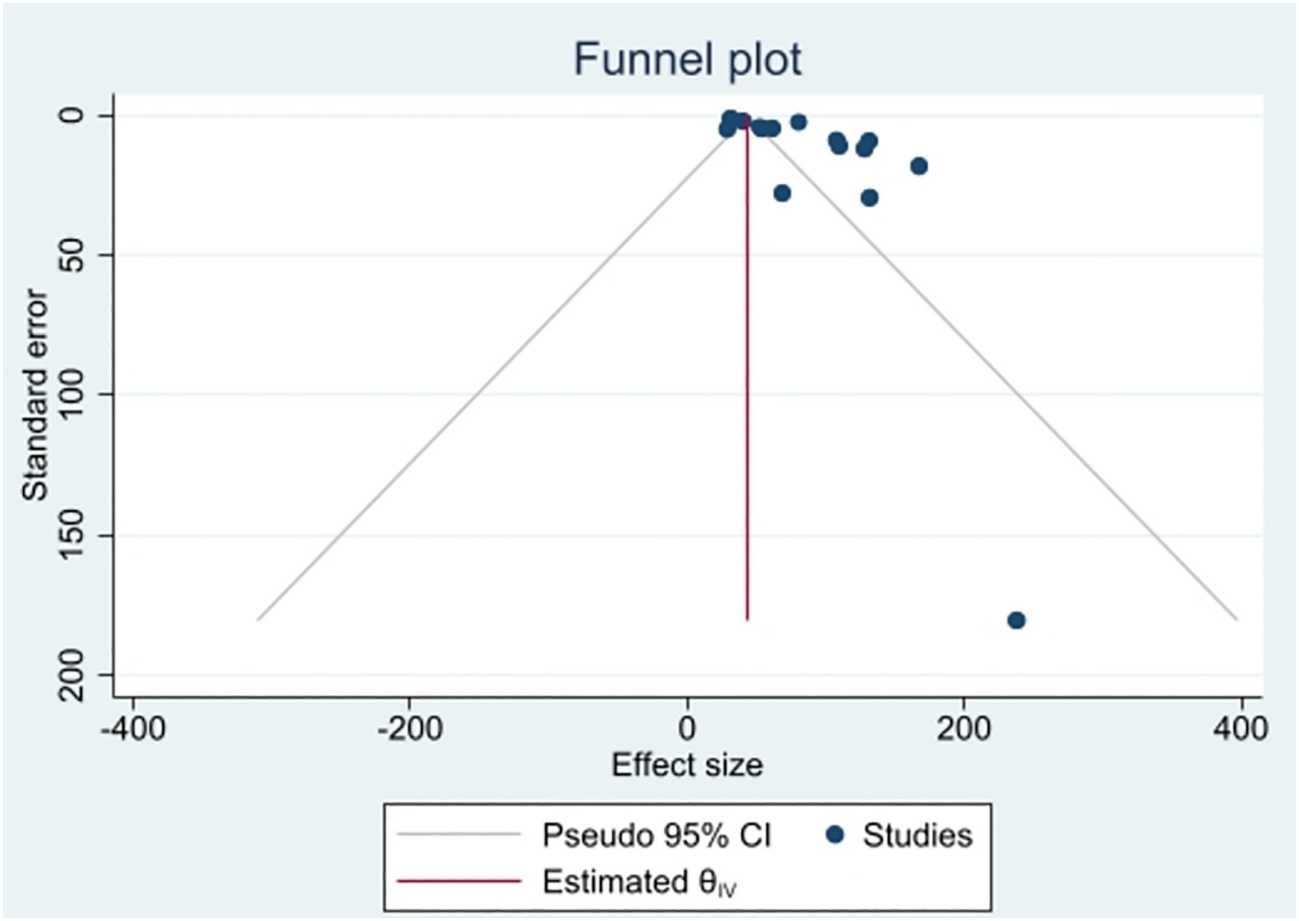
Funnel plot showing publication bias of studies included for meta-analysis on the birth prevalence of neural tube defects in Ethiopia, 2023.

Fifteen articles were included in the meta-analysis to estimate the prevalence of neural tube defects in Ethiopia. A total of 611,354 newborns and children under 17 years of age were included in the analysis. The reported sample size ranged from the minimum of 84 participants in Addis Ababa [44] to a maximum of 319,776 in the Addis Ababa and Amhara study [43]. The pooled birth prevalence of neural tube defects estimated using a random-effects model was 83.40 (95% CI: 60.78, 106.02) per 10,000 births (Fig 2). There was significant heterogeneity (I^2^ = 99.08%) among the included studies as well as significant evidence of publication bias identified by the funnel plot (Fig 3). The Egger’s test statistic for estimating birth prevalence was 2.37 (P = 0.0178), indicating some evidence of small-study effects. To adjust for this bias, we performed a trim and fill meta-analysis. In the fill meta-analysis, we looked at sixteen studies (1 article was included in fifteen studies). The random-effects model estimated the birth prevalence of neural tube defects to be 82.79 (95% CI: 60.25, 105.33) per 10,000 births (S1 Fig).

### Pooled prevalence of anencephaly in Ethiopia

**Fig 4:**
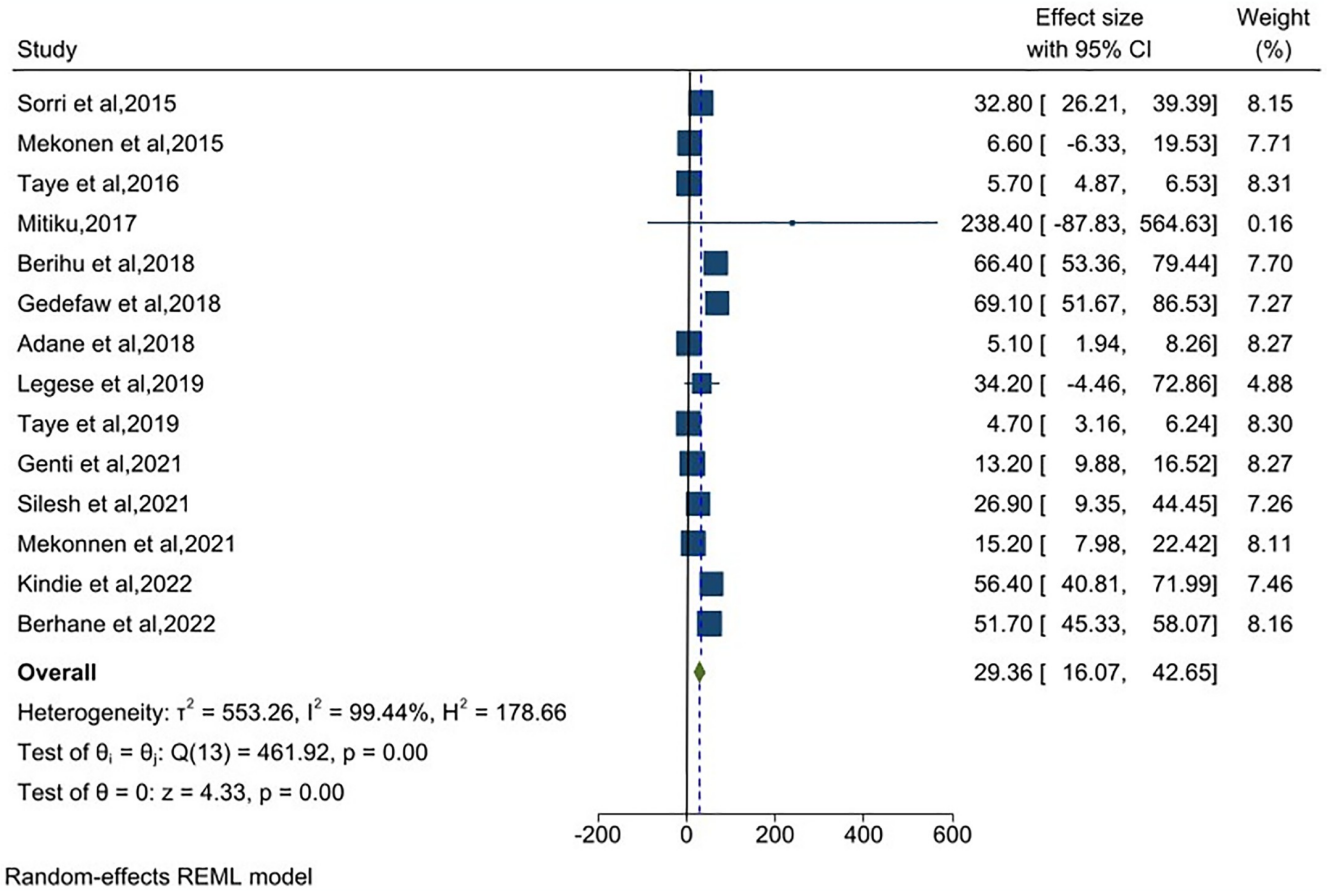
Meta-analysis, the prevalence of anencephaly per 10,000 births in Ethiopia, 2023.

Fourteen articles were included in the meta-analysis to estimate the share of anencephaly from total neural tube defect. The random-effects model estimated the pooled prevalence of anencephaly in Ethiopia was 29.36 (95%CI: 16.07, 42.65) per 10,000 births with (I^2^ = 99.44%) (Fig 4).

### Pooled prevalence of spinal bifida in Ethiopia

**Fig 5:**
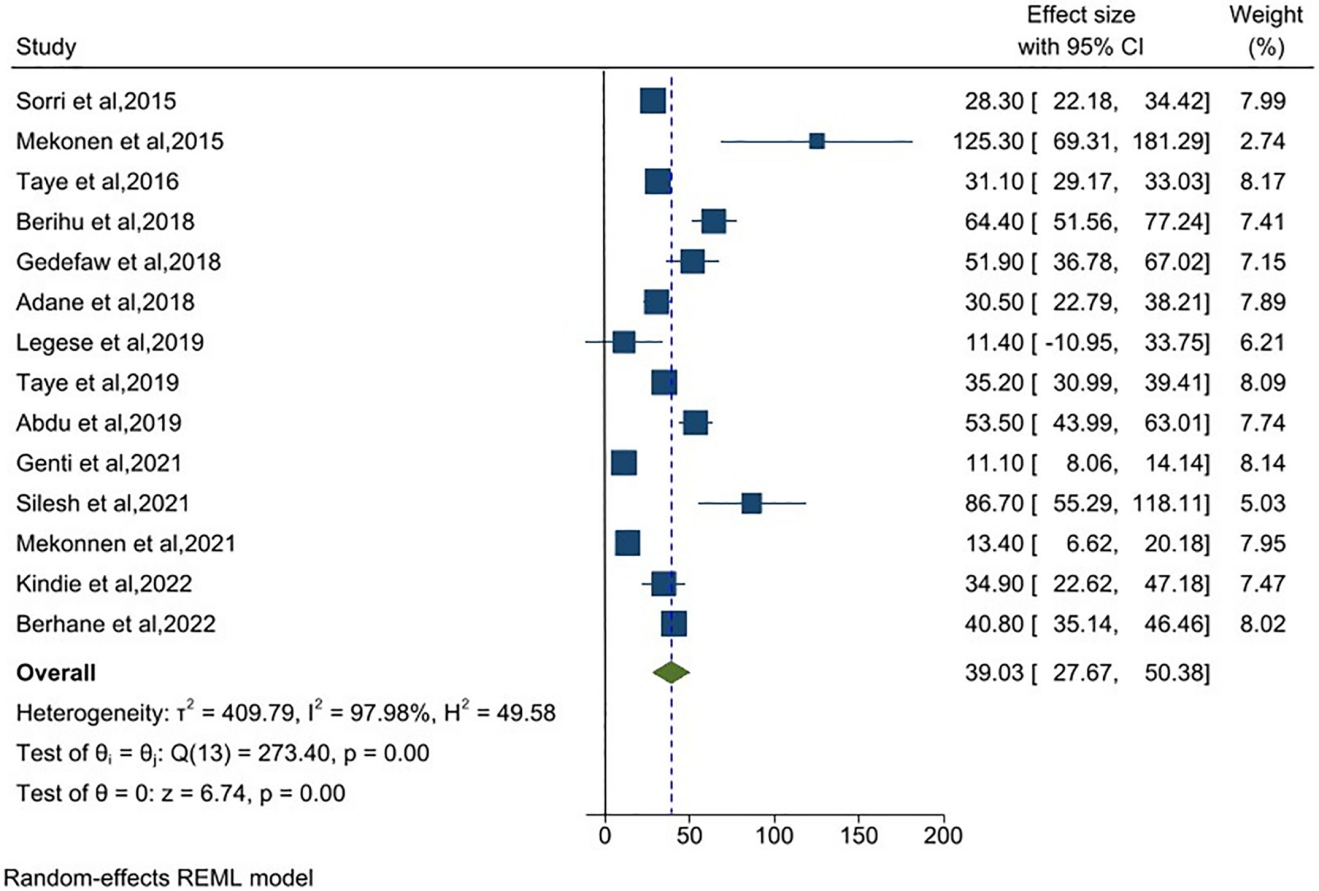
Meta-analysis, the prevalence of spina bifida per 10,000 births in Ethiopia, 2023.

Fourteen articles were included in the meta-analysis to estimate the share of spina bifida in total neural tube defects. The result of the random-effect meta-analysis estimated the pooled prevalence of spinal bifida type of NTDs in Ethiopia was 39.03 (95%CI: 27.67, 50.38) per 10,000 births (I^2^ = 97.98%) (Fig 5).

### Pooled prevalence of encephalocele in Ethiopia

**Fig 6:**
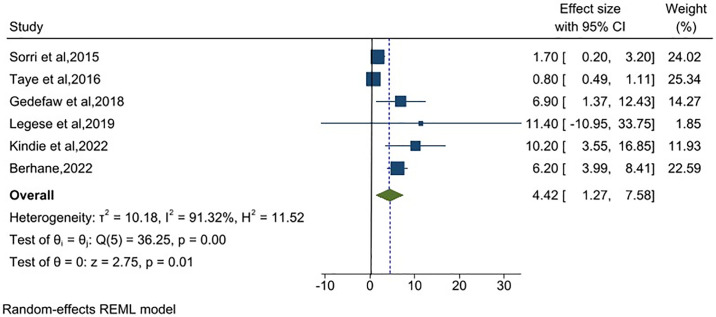
Meta-analysis, the prevalence of encephalocele per 10,000 births in Ethiopia, 2023.

Pooled odds ratios from six studies indicated were included in the meta-analysis to estimate the contribution of encephalocele to NTDs in Ethiopia. The random-effects model showed the pooled prevalence of encephalocele in Ethiopia was 4.42 (95% CI: 1.27, 7.58) per 10,000 births (I^2^ = 91.32%) (Fig 6).

### Sub-group analysis of the prevalence of NTDs

**Fig 7:**
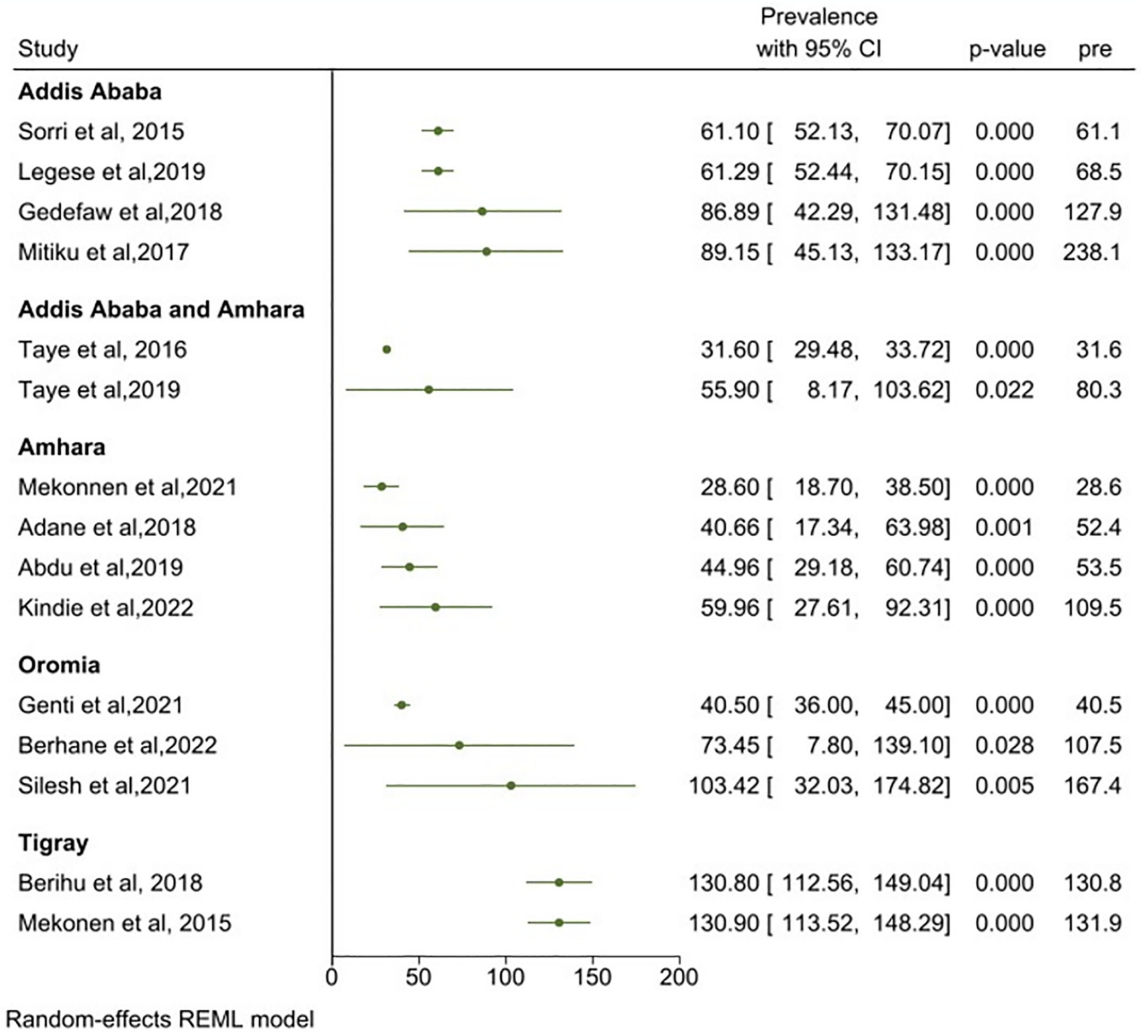
Meta-cumulative analysis showing effect of neural tube defects in relation to regional states of Ethiopia 2023.

The subgroup analysis by regional states and birth outcomes showed that, the highest and lowest prevalence rates were 130.9 (95% CI: 113.52, 148.29) in Tigray and 28.60 (95% CI: 18.70, 38.50) per 10,000 births in Amhara regional states (Fig 7). The prevalence of neural tube defects in both live births and stillbirths was 87.80 (95% CI: 62.61, 113.00) per 10,000 births (I^2^ = 98.08) (S2 Fig), and for NTDs in live births only was 59.04 (95% CI: 11.74, 106.33) per 10,000 births (I^2^ = 99.38) (S3 Fig).

### Determinants of neural tube defects in Ethiopia

In this review, socio-demographic, maternal reproductive factors, and health care factors were assessed for neural tube defects.

In this meta-analysis, taking folic acid supplements and planning pregnancy had a protective association for NTDs, whereas women with a stillbirth history, use of any drugs during pregnancy, exposure to radiation, and pesticides were risk factors significantly associated with neural tube defects. However, sex of the newborn (POR, 0.80; 95% CI: 0.53–1.19), maternal age (POR, 0.60; 95% CI: 0.24–1.53), residence (POR, 1.81; 95% CI: 0.64–5.08), maternal alcohol consumption (POR, 1.13; 95% CI: 0.55–2.32), and passive cigarette smoking (POR, 1.12; 95% CI: 0.26–4.88) were not significantly associated with neural tube defects in Ethiopia.

Women who took folic acid supplements had a 71% reduced risk of giving birth to a newborn with NTDs compared to their counterparts (POR, 0.29; 95% CI: 0.19, 0.45). Moderate heterogeneity (I^2^ = 64.33%; P<0.001) was detected among the included studies, and as a result, a random-effects meta-analysis model was computed. Women who had planned their pregnancy were 69% less likely to have a newborn with neural tube defects than women who had an unplanned pregnancy (POR, 0.31, 95% CI: 0.22, 0.43). There was no heterogeneity detected (I^2^ = 0%; P = 0.70) in this analysis. Among four studies, women who had a previous stillbirth history were 5.65 times more likely to have a newborn with neural tube defects than women who had no previous stillbirth history (POR, 5.65, 95% CI: 1.35–23.72). High heterogeneity (I^2^ = 83.69%; P = <0.001) was detected among the included studies, and as a result, a random-effects meta-analysis model was executed. Pooled odds ratios from 6 studies indicated that women who used any drugs during pregnancy were 2.54 times more likely to give birth to newborns with neural tube defects than non-drug users (POR, 2.54; 95%CI: 1.19, 5.42). High heterogeneity (I2 = 81.33%, P = <0.001) was detected among the included studies; thus, the random-effect meta-analysis model was performed.

Another significantly associated factors with NTDs were radiation and pesticide exposures. The study findings indicated that women who have been exposed to radiation were 3.85 times more likely to have a newborn with a neural tube defect than women who have not been exposed (POR, 3.85, 95% CI: 1.74, 8.51). Low heterogeneity (I^2^ = 6.97%; P = 0.39) was detected among the included studies; for this reason, the fixed-effect meta-analysis model was computed. Likewise, women who had pesticide exposure during pregnancy were 2.70 times more likely to have a newborn with NTDs (POR, 2.70; 95%CI: 1.63, 4.49). In this factor analysis, heterogeneity was not detected (I^2^ = 0%; P = 0.67 among the included studies). Hence, a fixed-effect meta-analysis model was used to do the final analysis (Table 3).

**Table 3:** Summary of determinants of neural tube defects among newborns in Ethiopia, 2023.

Author, year	Case		Control		I^2^ (%)	POR (95% CI)	P-value
	Yes	No	Yes	No			
**Folic acid supplement intake**							
Gedefaw et al,2018	15	68	96	154		0.35(0.19,0.65)	
Berihu et al,2019	9	36	196	376		0.48(0.23,1.02)	
Atlaw et al,2019	5	227	37	193		0.11(0.04,0.30)	
Tadesse et al,2020	28	129	99	125		0.27(0.17,0.45)	
Gashaw et al,2021	31	124	50	38		0.19(0.11,0.34)	
Mulu et al,2021	39	113	88	141		0.55(0.35,0.87)	
Tesfaye et al,2022	0	24	60	96		0.03(0.00,0.55)	
**Overall pooled**					64.33	0.29(0.19,0.45)	<0.001
**Planned pregnancy**							
Tesfaye et al,2021	40	100	20	20		0.40(0.19,0.82)	
Gashaw et al,2021	52	141	29	21		0.27(0.14,0.51)	
Mulu et al,2022	45	164	82	90		0.30(0.19,0.47)	
**Overall pooled**					0.00	0.31(0.22,0.43)	<0.001
**Previous still birth**							
Berihu et al,2019	14	2	112	306		19.13(4.28,85.48)	
Atlaw et al,2019	3	41	39	379		0.71(0.21,2.40)	
Edris et al,2020	10	19	14	377		14.17(5.57,36.04)	
Gashaw et al,2021	12	5	69	157		5.46(1.85,16.10)	
**Overall pooled**					83.69	5.65(1.35,23.72)	<0.001
**Use any drugs during pregnancy**							
Gedefaw et al,2018	22	23	89	249		2.68(1.42,5.4)	
Atlaw et al,2019	9	59	33	361		1.67(0.76,3.66)	
Abebe et al,2021	14	10	27	72		3.73(1.48,9.41)	
Mulu et al,2022	94	100	33	154		4.39(2.74,7.02)	
Edris et al,2020	9	33	15	363		6.60(2.68,16.23)	
Tesfay et al,2021	1	17	59	103		0.10(0.01,0.79)	
**Overall pooled**					81.33	2.54(1.19,5.42)	<0.001
**Radiation exposures**							
Berihu et al,2019	5	2	200	410		5.13(0.99,26.65)	
Atlaw et al,2019	2	9	40	411		2.28(0.48,10.93)	
Edris et al,2020	4	10	20	386		7.72(2.23,26.77)	
Tesfaye et al,2021	2	3	58	117		1.34(0.22,8.27)	
**Overall pooled**					6.97	3.85 (1.74, 8.51)	<0.001
**Pesticide exposures**							
Berihu et al,2019	5	2	200	410		5.13(0.99,26.65)	
Atlaw et al,2019	1	2	41	418		5.10(0.45,57.43)	
Gashaw et al,2021	15	11	66	151		3.12(1.36,7.16)	
Tesfaye et al,2021	18	21	42	99		2.02(0.98,4.17)	
**Overall pooled**					0.00	2.70 (1.63, 4.49)	<0.001

## Discussion

In this systematic review and meta-analysis, the prevalence and determinants of NTDs in Ethiopia were reviewed and analyzed. The pooled prevalence of neural tube defects in Ethiopia was 83.40 per 10,000 births. This prevalence is higher than systematic reviews conducted by Oumer et al. for Africa, which found a neural tube defect prevalence of 21.42 per 10,000 births [6], a summary review of 11.7 per 10,000 births in low and middle-income countries [61], and a study by Bitew et al. in Ethiopia, which found 63.3 per 10,000 births [62]. Possible explanations for the higher prevalence of NTDs in this study could be related to differences in maternal socio-economic status, periconceptional folic acid supplementation, and other disparities in the study participants.

Concerning subtypes of neural tube defects, the prevalence of anencephaly in Ethiopia was 29.36 per 10,000 births. The pooled prevalence in this study is higher than the 21.1 in India [63], 4.92 in China [64] and 19.11 cases in Africa [65] per 10,000 births. Likewise, in this systematic review, the estimated prevalence of spina bifida was 39.03 per 10,000 births, higher than studies in China [66] and overall in Africa [65] (6.25 and 29.67 per 10,000 births, respectively). However, the prevalence of encephalocele in Ethiopia was 4.42 per 10,000 births, which is in line with the prevalence in Nepal [67]. The discrepancy in the subtypes of neural tube defects might be related to differences in the diagnosing abilities of health professionals and a lack of access to ultrasound screening during pregnancy.

The meta-analysis result showed that women who took folic acid supplementation at any time within the pregnancy period were 71% less likely to have newborns with neural tube defects than their counterparts. The finding is supported by studies that indicate folic acid supplementation during the periconceptional period reduces the risk of neural tube defects [68–70]. Folate-preventable NTDs are high due to inadequate counselling about the benefits of folic acid for the prevention of congenital malformation and a lack of periconceptional folic acid supplementation. Also this review found that women who have a stillbirth history were nearly six times more likely to have a newborn with neural tube defects than women who had no previous stillbirths. A study by Glinianaia et al. indicated that repeated stillbirths and/or previous miscarriages increase the risk of neural tube defects [71].

Most NTDs in Ethiopia were associated with stillbirths and the number we report may be an underestimation because of unreported stillbirths and miscarriages in home deliveries. However, women who have planned a pregnancy are 69% less likely to have a newborn with neural tube defects than women who have an unplanned pregnancy. This data is strengthened by a study that found a link between key prevention measures for NTD and pregnancy planning [72]. Women who use any drugs during pregnancy were three times more likely to give birth to newborns with neural tube defects than non-drug users. Studies have found a link between NTDs and medications, especially anti-epileptic drugs and the most commonly used over-the-counter medications in the community, such as psychoactive drugs, antibiotics, and non-steroidal anti-inflammatory drugs (NSAIDs) [19, 20, 73–75].

Other factors significantly associated with NTDs were radiation and pesticide exposures. Women who had been exposed to radiation were four times more likely to have newborns with neural tube defects than women who have not been exposed. The findings indicate that exposure to ionizing radiation during neurulation resulted in neural tube defects [76, 77]. The odds of having a neural tube defect affect a newborn among pesticide-exposed women were three times higher than their counterparts. This finding is supported by evidence from other regions of the world, which demonstrates that maternal pesticide exposure is linked to the incidence of NTDs [78–80]. Pesticide exposure relates to the proximity of the cultivating farmland to the residence of the women, which leads to contamination.

### Strength and limitations of the review

This systematic review and meta-analysis provide insight on neural tube defects and determinants. The pooled estimate from twenty-five studies in Ethiopia will provide remarkable evidence for policymakers in the field of maternal and child care services. The current review has some limitations in that most of the studies were done in facilities and underestimate the prevalence of under-reporting of miscarriage and stillbirths from home delivery in the community. Furthermore, data are unavailable from some regions of Ethiopia because of lack of primary researches.

## Conclusion

The pooled birth prevalence of NTDs in Ethiopia was found to be high. In this meta-analysis, women’s intake of folic acid supplements and planned pregnancy were associated with reduced prevalence NTDs, while stillbirth history, use of certain drugs during pregnancy, and exposure to radiation and pesticides during pregnancy were significantly associated determinants of NTDs. We would like to remind policymakers that the pooled birth prevalence estimates may be underestimated due to insufficient evidence across the various parts of Ethiopia among other reasons. The high pooled estimate supports the need for policy decisions supporting prevention efforts in Ethiopia. Effective prevention interventions, especially focusing on periconceptional folic acid supplementation as well as folate fortification, should be prioritized alongside nutrition education, maternal health care, and environmental safety measures. Furthermore, the scarcity of data on neural tube defects highlights the need for more primary data with a broader scope of research evidence to identify the true burden of neural tube defects and to support preventive measures in low-income countries, including Ethiopia.

## Supporting information

S1 Fig Trim and fill meta-analysis for the birth prevalence of neural tube defects in Ethiopia. (TIF)

S2 Fig Live birth and Still birth outcomes of neonates with neural tube defects for meta-analysis on the birth prevalence of neural tube defects in Ethiopia. (TIF)

S3 Fig Live birth outcomes of neonates with neural tube defects for meta-analysis on the birth prevalence of neural tube defects in Ethiopia. (TIF)

S1 Table PRISMA 2020 checklist reporting the findings of the systematic review and meta-analysis on birth prevalence and determinants of neural tube defects among newborns in Ethiopia. (PDF)

S2 Table Critical appraisal checklists for all study designs on systematic review and meta-analysis for the birth prevalence and determinants of neural tube defects among newborns in Ethiopia. (PDF)

S3 Table Extracted data for included studies in systematic review and meta-analysis for the birth prevalence and determinants of neural tube defects among newborns in Ethiopia. (PDF)

S1 Appendix Database search strategy for systematic review and meta-analysis on birth prevalence and determinants of neural tube defects among newborns in Ethiopia. (PDF)

S2 Appendix All studies identified in the literature search for birth prevalence and determinants of neural tube defects in Ethiopia from April 14 to August 24, 2023. (PDF)
