# Correction: Birth prevalence and determinants of neural tube defects among newborns in Ethiopia: A systematic review and meta-analysis

**DOI:** 10.1371/journal.pone.0342989

**Published:** 2026-02-13

**Authors:** Beminet Moges Gebremariam, Dejene Hailu, Barbara J. Stoecker, Afework Mulugeta

There are errors in the author affiliations. The correct affiliations are as follows:

Beminet Moges Gebremariam^1^, Dejene Hailu^2^, Barbara J. Stoecker^3^, Afework Mulugeta^4^

**1** School of Nutrition, Food Science and Technology, College of Agriculture, Hawassa University, Hawassa, Ethiopia, **2** School of Public Health, College of Medicine and Health Sciences, Hawassa University, Hawassa, Ethiopia, **3** Department of Nutritional Sciences, Oklahoma State University, Stillwater, Oklahoma,United States of America, **4** Department of Public Health Sciences, Mekelle University, Mekelle, Ethiopia.
