# Supplementary figures and images for: Birth prevalence and determinants of neural tube defects among newborns in Ethiopia: A systematic review and meta-analysis
Source: PLoS One. 2025 Jan 2;20(1):e0315122. doi: 10.1371/journal.pone.0315122 (PMC11695007; doi:10.1371/journal.pone.0315122)

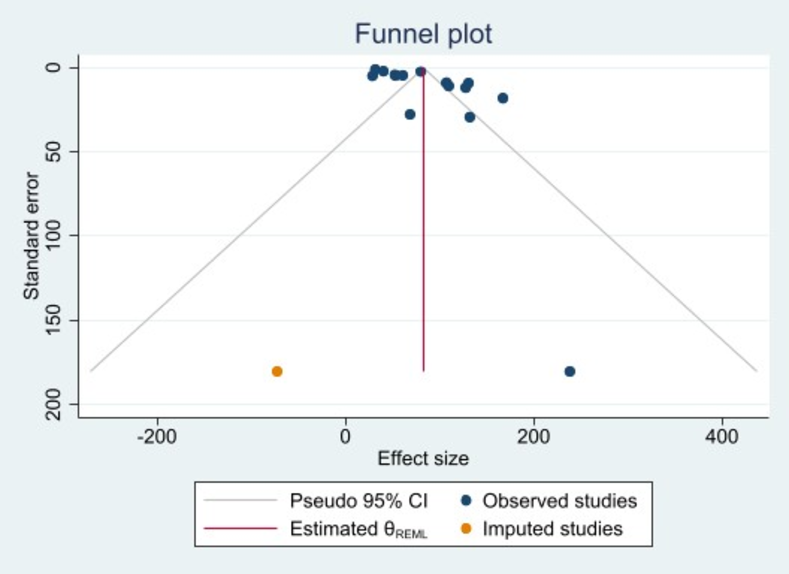

Supplement: S1 Fig — (TIF) [file pone.0315122.s001.tif]

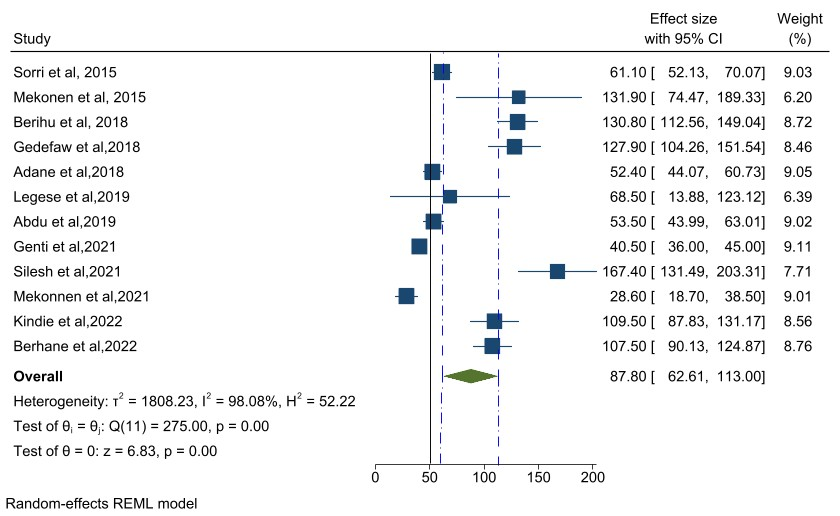

Supplement: S2 Fig — (TIF) [file pone.0315122.s002.tif]

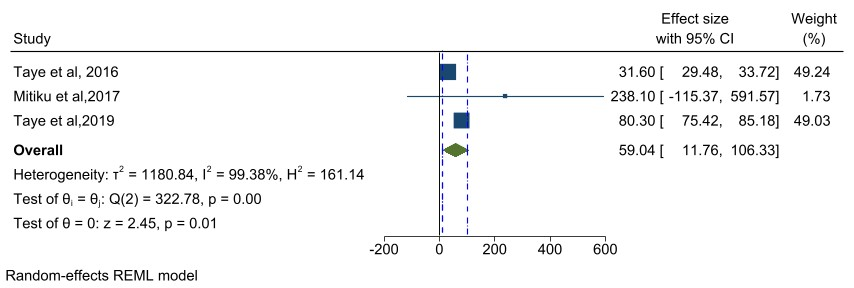

Supplement: S3 Fig — (TIF) [file pone.0315122.s003.tif]
