# Supplementary material for: Birth prevalence and determinants of neural tube defects among newborns in Ethiopia: A systematic review and meta-analysis
Source: PLoS One. 2025 Jan 2;20(1):e0315122. doi: 10.1371/journal.pone.0315122 (PMC11695007; doi:10.1371/journal.pone.0315122)
Supplement: S2 Table — (PDF) [file pone.0315122.s005.pdf]

Table.1.1 Critical appraisal for cross-sectional studies 2023

Author Name, Year	Q1			Q2			Q3			Q4			Q5			Q6			Q7			Q8			Risk of bias
	Y	N	U	Y	N	U	Y	N	U	Y	N	U	Y	N	U	Y	N	U	Y	N	U	Y	N	U	
Sorri et al, 2015	1			1			1				0			0			0		1			1			(5)= Medium
Taye et al, 2016	1				0		1			1				0			0		1			1			(5)=Medium
Mitiku et al,2017	1			1			1			1			0				0			0			0		(4)=High
Berihu et al, 2018	1			1			1			1				0			0		1			1			(6)=Medium
Gedefaw et al,2018		0			0		1			1			1			1			1			1			(6)=Medium
Adane et al,2018		0			0		1				0		1			1			1			1			(5)=Medium
Legese et al,2019	1			1			1			1				0			0		1			1			(6)=Medium
Taye et al,2019	1			1			1			1			1			1			1			1			(8)=Low
Abdu et al,2019	1			1			1			1				0			0					1			(6)=Medium
Genti et al,2021	1			1			1			1				0			0		1			1			(6)=Medium
Silesh et al,2021	1			1			1			1				0			0		0			1			(5)= Medium
Mekonnen et al,2021	1			1			1			1			1			1			1			1			(8)= Low
Kindie et al,2022	1			1			1			1				0			0		1			1			(6)= Medium
Edris et al,2020	1			1			1			1			1			1			1			1			(8)= Low

NB. ***Y=yes, N=no, U=unclear, low risk (≥ 7), medium risk (5 to 6) and high risk (≤ 4)***

Q1: Were the criteria for inclusion in the sample clearly defined?

Q2: Were the study subjects and the setting described in detail?

Q3: Was the exposure measured in a valid and reliable way?

Q4: Were objective, standard criteria used for measurement of the condition?

Q5: Were confounding factors identified?

Q6: Were strategies to deal with confounding factors stated?

Q7: Were the outcomes measured in a valid and reliable way?

Q8: Was appropriate statistical analysis used?

Table1.2 Critical appraisal for case-control studies 2023

Author Name	Q1			Q2			Q3			Q4			Q5			Q6			Q7			Q8			Q9			Q10			Risk of Bias
	Y	N	U	Y	N	U	Y	N	U	Y	N	U	Y	N	U	Y	N	U	Y	N	U	Y	N	U	Y	N	U	Y	N	U	
Berihu et al,2019	1				0		1			1			1				0			0			1		1				0		(6)=Medium
Gedefaw et al,2018	1				0		1			1			1			1			1			1			1			1			(9)=Low
Atlaw et al,2019	1				0			0					1			1			1			1			1			1			(8)=Low
Tadesse et al,2020	1				0		1			1			1			1			1			1			1			1			(9)=Low
Abebe et al,2021	1				0		1			1			1			1			1			1				0		1			(8)=Low
Tesfaye et al,2021	1				0		1			1			1			1			1			1			1			1			(9)=Low
Gashaw et al,2021	1				0		1			1			1			1			1			1			1			1			(9)=Low
Getinet et al,2021	1				0		1			1			1			1			1			1			1			1			(9)=Low
Mulu et al,2022	1				0		1			1			1			1			1			1			1			1			(9)=Low

NB. ***Y=yes, N=no, U=unclear, high risk (< 5), medium risk (6 to 7) and low risk (≥8)***

Q1: Were the groups comparable other than the presence of disease in cases or the absence of disease in controls?

Q2: Were cases and controls matched appropriately?

Q3: Were the same criteria used for identification of cases and controls?

Q4: Was exposure measured in a standard, valid and reliable way?

Q5: Was exposure measured in the same way for cases and controls?

Q6: Were confounding factors identified?

Q7: Were strategies to deal with confounding factors stated?

Q8: Were outcomes assessed in a standard, valid and reliable way for cases and controls?

Q9: Was the exposure period of interest long enough to be meaningful?

Q10: Was appropriate statistical analysis used?

Table 1.3 Critical appraisal for cohort studies 2023

Author Name	Q1			Q2			Q3			Q4			Q5			Q6			Q7			Q8			Q9			Q10			Q11			Risk of Bias
	Y	N	U	Y	N	U	Y	N	U	Y	N	U	Y	N	U	Y	N	U	Y	N	U	Y	N	U	Y	N	U	Y	N	U	Y	N	U	
Mekonen et al, 2015		0			0			0			0			0		1			1			1				0		1				0		(4)=High
Berhane et al,2022		0			0			0			0			0		1			1			1			1			1			1			(6)= Medium

NB. *Y=yes, N=no, U=unclear, high risk (≤5), medium risk (6 to 7) and low risk (≥8)

Q1: Were the two groups similar and recruited from the same population?

Q2: Were the exposures measured similarly to assign people to both exposed and unexposed groups?

Q3: Was the exposure measured in a valid and reliable way

Q4: Were confounding factors identified?

Q5: Were strategies to deal with confounding factors stated?

Q6: Were the groups/participants free of the outcome at the start of the study (or at the moment of exposure)?

Q7: Were the outcomes measured in a valid and reliable way?

Q8: Was the follow up time reported and sufficient to be long enough for outcomes to occur?

Q9: Were strategies to address incomplete follow up utilized?

Q10: Was follow up complete, and if not, were the reasons to loss to follow up described and explored?

Q11: Was appropriate statistical analysis used?
