# Supplementary material for: Birth prevalence and determinants of neural tube defects among newborns in Ethiopia: A systematic review and meta-analysis
Source: PLoS One. 2025 Jan 2;20(1):e0315122. doi: 10.1371/journal.pone.0315122 (PMC11695007; doi:10.1371/journal.pone.0315122)
Supplement: S3 Table — (PDF) [file pone.0315122.s006.pdf]

Extracted Studies

1) Data extraction was performed by two independent reviewers (BMG and DH) between August 28 and September 16, 2023, using a standardized data extraction tool developed by JBI, and the data were subsequently sorted.
2) We confirm that all studies included in the review fulfilled predefined inclusion criteria.
3) All data retrieved for this systematic review and meta-analysis, including author, publication year, region, study design, quality assessment, sample size, prevalence, and study quality assessment, were used to replicate the analyses.

I. Characteristics of included study for birth prevalence of NTDs among newborns in Ethiopia

Study	Publication Year	Region	Study design	Quality Ass.	Sample	NTDs Prevalence per 10,000	Link DOI/ [URL]
Sorri	2015	Addis Ababa	Cross-sectional	5	28961	61.1	https://emjema.org/index.php/EMJ/article/view/120/pdf_3
Mekonen	2015	Tigray	Prospective cohort	4	1516	131.9	https://doi.org/10.1186/s12884-015-0507-2.
Taye	2016	Addis Ababa and Amhara	Cross-sectional	5	319776	31.6	https://doi.org/10.1371/journal.pone.0161998.
Mitiku	2017	Addis Ababa	Cross-sectional	4	84	238.1	https://etd.aau.edu.et/items/3e6736f4-213f-4316-9997-89d55c5813d9 Unpublished
Berihu	2018	Tigray	Cross-sectional	6	14903	130.8	https://doi.org/10.1371/journal.pone.0206212
Gedefaw	2018	Addis Ababa	Cross-sectional	6	8677	127.9	https://doi.org/10.1155/2018/4829023
Adane	2018	Amhara	Cross-sectional	5	19650	52.4	https://www.ajol.info/index.php/ejhd/article/view/178807

Legese	2019	Addis Ababa	Cross-sectional	6	876	68.5	https://etd.aau.edu.et/server/api/core/bitstreams/a32290f2-e827-4129-a23b-4a4cf05a9017/content Unpublished
Taye	2019	Addis Ababa and Amhara	Cross-sectional	8	76201	80.3	https://doi.org/10.1186/s12887-019-1596-2.
Abdu	2019	Amhara	Cross-sectional	6	22624	53.5	https://www.ajol.info/index.php/ejhd/article/view/190126
Genti	2021	Oromia	Cross-sectional	6	45951	40.5	https://doi.org/10.11604/pamj.2021.40.248.25286
Silesh	2021	Oromia	Cross-sectional	5	3346	167.4	https://doi.org/10.2147/PHMT.S293285.
Mekonnen	2021	Amhara	Cross-sectional	8	11177	28.6	https://doi.org/10.1038/s41598-021-90387-0.
Kindie	2022	Amhara	Cross-sectional	6	8862	109.5	https://doi.org/10.1371/journal.pone.0261177
Berhane	2022	Oromia	Retrospective cohort	6	48750	107.5	https://doi.org/10.1371/journal.pone.0264005.

II. Characteristics of included study for determinants of NTDs among newborns in Ethiopia

Study	Publication Year	Region	Study design	Quality Ass.	Sample	Factors	OR (95%CI)	Link: DOI/ [URL]
Berihu	2019	Tigray	Case-control	6	617	Maternal age < 35 yrs	2.46 (1.33,4.53)	https://doi.org/10.1016/j.braindev.2018.07.013.
						Folic acid tablets	2.15 (1.02,4.54)	
						Previous history of stillbirth	19.1 (4.28,85.48)	
						Exposure to radiation	5 (0.150,166.60)	
						Maternal alcohol consumption	10.3(1.19,88.50)	
						Exposure to pesticide	5 (0.150,166.60)	
						Unplanned pregnancy	1.97 (1.38,2.82)	
Gedefaw	2018	Addis Ababa	Case-control Part of a study	9	333	Maternal age < 35yrs	2(0.73,5.47)	https://doi.org/10.1155/2018/4829023
						Folic acid tablets	0.47 (0.23,0.95)	
						Previous history of stillbirth	0.49(0.10,2.35)	
						Planned pregnancy	0.47 (0.24,0.92)	
Atlaw	2019	Oromia	Case-control	8	462	Maternal age |<35 yrs	4.77 (1.10-20.66)	https://www.omicsonline.org/open-access/neural-tube-defect-and-associated-factors-in-bale-zone-hospitals-southeast-ethiopia-109137.html
						Folic acid tablets	0.09 (.031, 285)	
						Previous history of stillbirth	1.41 (.42,4.75)	
						Exposure to radiation	0.44 (.09, 2.08)	

						Alcohol consumption	0.79 (.32,1.98)	
						Exposure to pesticide	0.19 (.02,2.2)	
Tadesse	2020	Amhara	Case-control	9	400	Rural residence	1.78(1.02,3.11)	https://doi.org/10.1155/2020/5635267.
						Folic acid tablets	0.37(0.21,0.65)	
						Unplanned pregnancy	0.94 (0.54,1.66)	
						Never took any substance	0.42 (0.21,0.88)	
Edris	2020	Oromia	Cross-sectional	8	420	Maternal age< 35 yrs	3.84 (2.1,10.7)	https://doi.org/10.1177/2333794X20974218.
						Urban residence	0.48 (0.2,3.7)	
						Radiation exposure	5.01 (1.6,14.3)	
						AEDs drug intake	4.75 (1.5,16.2)	
Abebe	2021	Amhara	Case-control	8	123	Maternal age less than 35 years old	0.13(0.02,0.72)	https://doi.org/10.2147/PHMT.S332561.
						Any drug intake	3.73(1.48,9.41)	
Tesfaye	2021	Addis Ababa	Case-control	9	180	Maternal age < 35yrs	33.7 (2.53,448.5)	https://doi.org/10.1016/j.ijans.2021.100318.
						Planned pregnancy	0.41 (0.08,2.30)	
						Passive cigarette smoking	1.0(0.23,9.91)	
Gashaw	2021	Amhara	Case-control	9	243	Family annual income < 24000ETB	3.73(1.35,10.26)	https://doi.org/10.1371/journal.pone.0250719.
						History of still birth	3.63(1.03,12.2)	

						History of abortion	6.15(2.63,18.56)	
						Pesticides exposure	5.34(1.77,16.05)	
						preconception care	0.14(0.05,0.39)	
						Folic acid tablets	0.16 (0.07,0.33)	
Getinet	2021	Oromia	Case-control	9	219	Rural residence	7.99 (3.99,16.03)	https://www.researchgate.net/publication/364113720
Mulu	2022	Amhara	Case-control	9	381	Intake of medication during pregnancy	1.83(1.08,3.08)	https://doi.org/10.1371/journal.pone.0261177.
						Mothers who did not take a balanced diet	13.46(7.83,23.13)	
						Folic acid supplementation	1.71 (1.01,2.94)	
