# Supplementary material for: Birth prevalence and determinants of neural tube defects among newborns in Ethiopia: A systematic review and meta-analysis
Source: PLoS One. 2025 Jan 2;20(1):e0315122. doi: 10.1371/journal.pone.0315122 (PMC11695007; doi:10.1371/journal.pone.0315122)
Supplement: S1 Appendix — (PDF) [file pone.0315122.s007.pdf]

Database search strategy

The databases used to search for studies were stated below:

I. PubMed Searching Methods

Sr No	Searching Term	Number of articles
1	(“birth prevalence” OR “burden”) AND (“neural tube defects”) AND (newborns OR neonates) AND (Ethiopia OR Regions)	454
2	("neural tube defect") AND ("associated factors" OR "determinants" OR "risk factors") AND ("Ethiopia" OR “Regional states”)	213
	Total	667

- ✓ The Boolean operators "AND" and "OR" were employed in conjunction with the searching terms

II. Science Direct and Cochrane Library

Sr No	Databases	Searching Term/MeSH term	Number of articles
1	Science Direct	prevalence of neural tube defects and associated factors among neonates in Ethiopia	160
2	Cochrane Library	neural tube defects and associated factors in Ethiopia	2
	Total		162

MeSH: Medical Subject Headings

III. Additional databases searched

The other databases' article searches used interchangeable key search terms (prevalence, neural tube defects, associated factors/determinants/risk factors, newborns, and Ethiopia).

Sr No	Databases	Number of articles
1	Google Scholar	45
2	Research Gate	4
3	Google	38
	Total	87
