# Supplementary material for: Birth prevalence and determinants of neural tube defects among newborns in Ethiopia: A systematic review and meta-analysis
Source: PLoS One. 2025 Jan 2;20(1):e0315122. doi: 10.1371/journal.pone.0315122 (PMC11695007; doi:10.1371/journal.pone.0315122)
Supplement: S2 Appendix — (PDF) [file pone.0315122.s008.pdf]

All studies identified in the literature search for birth prevalence and determinants of neural tube defects in Ethiopia from April 14 to August 24, 2023.

Sr. No	Author, Year	Status	Reason for Inclusion/ Exclusion	URL
1.	Sorri et al. (2015).	Included	Fulfilled criteria	https://emjema.org/index.php/EMJ/article/view/120/pdf_3
2.	Mekonen et al. (2015).	Included	Fulfilled criteria	https://doi.org/10.1186/s12884-015-0507-2.
3.	Taye et al. (2016).	Included	Fulfilled criteria	https://doi.org/10.1371/journal.pone.0161998.
4.	Mitiku et al. (2017).	Included	Fulfilled criteria	https://etd.aau.edu.et/items/3e6736f4-213f-4316-9997-89d55c5813d9 *Unpublished*
5.	Berihu et al. (2018).	Included	Fulfilled criteria	https://doi.org/10.1371/journal.pone.0206212
6.	Gedefaw et al. (2018).	Included	Fulfilled criteria *Magnitude of NTDs part*	https://doi.org/10.1155/2018/4829023
7.	Adane et al. (2018).	Included	Fulfilled criteria	https://www.ajol.info/index.php/ejhd/article/view/178807
8.	Legese et al. (2019).	Included	Fulfilled criteria	https://etd.aau.edu.et/server/api/core/bitstreams/a32290f2-e827-4129-a23b-4a4cf05a9017/content *Unpublished*
9.	Taye et al. (2019).	Included	Fulfilled criteria	https://doi.org/10.1186/s12887-019-1596-2.
10.	Abdu et al. (2019).	Included	Fulfilled criteria	https://www.ajol.info/index.php/ejhd/article/view/190126
11.	Genti et al. (2021).	Included	Fulfilled criteria	https://doi.org/10.11604/pamj.2021.40.248.25286
12.	Silesh et al. (2021).	Included	Fulfilled criteria	https://doi.org/10.2147/PHMT.S293285.
13.	Mekonnen et al. (2021).	Included	Fulfilled criteria	https://doi.org/10.1038/s41598-021-90387-0.
14.	Kindie et al. (2022).	Included	Fulfilled criteria	https://doi.org/10.1371/journal.pone.0261177

15.	Berhane et al. (2022).	Included	Fulfilled criteria	https://doi.org/10.1371/journal.pone.0264005.
16.	Berihu et al. (2019).	Included	Fulfilled criteria	https://doi.org/10.1016/j.braindev.2018.07.013.
17.	Gedefaw et al. (2018).	Included	Fulfilled criteria *Case-control part*	https://doi.org/10.1155/2018/4829023
18.	Atlaw et al. (2019).	Included	Fulfilled criteria	https://www.omicsonline.org/open-access/neural-tube-defects
19.	Tadesse et al. (2020).	Included	Fulfilled criteria	https://doi.org/10.1155/2020/5635267.
20.	Edris et al. (2020).	Included	Fulfilled criteria	https://doi.org/10.1177/2333794X20974218.
21.	Abebe et al. (2021).	Included	Fulfilled criteria	https://doi.org/10.2147/PHMT.S332561.
22.	Tesfaye et al. (2021).	Included	Fulfilled criteria	https://doi.org/10.1016/j.ijans.2021.100318.
23.	Gashaw et al. (2021).	Included	Fulfilled criteria	https://doi.org/10.1371/journal.pone.0250719.
24.	Getinet et al. (2021).	Included	Fulfilled criteria	https://www.researchgate.net/publication/364113720
25.	Mulu et al. (2022).	Included	Fulfilled criteria	https://doi.org/10.1371/journal.pone.0261177.
26.	Atlaw, D., et al. (2021).	Excluded	Design difference, study area difference	https://doi.org/10.1186/s12884-021-03848-9
27.	Begashaw, B., et al. (2022).	Excluded	Research outcomes difference	https://doi:10.1017/jns.2022.32
28.	Dessie MA, et al. (2017).	Excluded	Research outcomes difference	https://doi.org/10.1186/s12884-017-1506-2
29.	Dixon, M., et al. (2019)	Excluded	Research outcomes difference	https://doi.org/10.1002/bdr2.1584
30.	Eshete, M., et al. (2017).	Excluded	Research outcomes difference	https://doi.org/10.1097/scs.0000000000003234
31.	Getahun, S., et al. (2021).	Excluded	Research outcomes difference	https://doi.org/10.1016/j.wneu.2021.01.098
32.	Kancherla, V., et al. (2021).	Excluded	Research outcomes difference	https://doi.org/10.1002/bdr2.1857
33.	Koning, M., et al. (2023).	Excluded	Design difference	https://doi.org/10.1007/s00381-023-05932-1
34.	Kucha, W., et al. (2022).	Excluded	Research outcomes difference	https://doi.org/10.3389/fnut.2022.873900
35.	Madrid, L., et al. (2023).	Excluded	Research outcomes not specific to Ethiopia	https://doi.org/10.1016/s2214-109x(23)00191-2

36.	Mulugeta, B., et al. (2022).	Excluded	Research outcomes difference	https://doi.org/10.1186/s12887-022-03212-6
37.	Tsehay, B., et al. (2019).	Excluded	Inability to include specific outcomes	https://doi.org/10.1186/s13104-019-4541-4
38.	Ssentongo, P., et al. (2022).	Excluded	Design difference, study area difference	https://doi.org/10.1186/s12883-022-02697-z
39.	Welderufael, A. L., et al. (2019).	Excluded	Research outcomes difference	https://doi.org/10.1016/j.braindev.2018.12.005
40.	Aarsland, S. J., et al. (2012).	Excluded	Unrelated topic	https://doi.org/10.4269/ajtmh.2012.12-0171
41.	Abayo, G., et al. (2021).	Excluded	Unrelated topic	https://doi.org/10.4314/ejhs.v31i4.18
42.	Abdissa, A., et al. (2011).	Excluded	Unrelated topic	https://pubmed.ncbi.nlm.nih.gov/21796912/
43.	Abebe, D., et al. (2012).	Excluded	Unrelated topic	https://pubmed.ncbi.nlm.nih.gov/22946292/
44.	Abebe, G. F., et al. (2022).	Excluded	Unrelated topic	https://doi.org/10.1186/s12884-022-05016-z
45.	Abeywardana, S., et al. (2010).	Excluded	Study area difference	https://doi.org/10.1111/j.1753-6405.2010.00565.x
46.	Abrahim, O., et al. (2015).	Excluded	Unrelated topic	https://doi.org/10.1371/journal.pone.0139024
47.	Abreha, S. K., et al. (2020).	Excluded	Unrelated topic	https://doi.org/10.1371/journal.pone.0235825
48.	Abreha, T., et al. (2017).	Excluded	Unrelated topic	https://doi.org/10.1371/journal.pmed.1002299
49.	Abu-Heija, A. T. (1994).	Excluded	Study area difference	https://doi.org/10.1111/j.1447-0756.1994.tb00490.x
50.	Abuye, C. and Y. Berhane (2007).	Excluded	Unrelated topic	https://doi.org/10.1186/1471-2458-7-316
51.	Abuye, C., et al. (2008).	Excluded	Unrelated topic	https://doi.org/10.4314/eajph.v5i3.38997
52.	Adane, K., et al. (2016).	Excluded	Unrelated topic	https://doi.org/10.1371/journal.pone.0149453
53.	Adane, M., et al. (2017).	Excluded	Unrelated topic	https://doi.org/10.1186/s41043-017-0085-1
54.	Adeleye, A. O.,et al. (2015).	Excluded	Study area difference	https://doi.org/10.1007/s00381-015-2718-2
55.	Adewuya, A. O., et al. (2014).	Excluded	Unrelated topic	https://doi.org/10.1111/jpc.12411
56.	Adinew, Y. M., et al. (2017).	Excluded	Unrelated topic	https://doi.org/10.1186/s12978-017-0434-y
57.	Agha, M. M., et al. (2013).	Excluded	Study area difference	https://doi.org/10.3390/ijerph10041312
58.	Agopian, A. J., et al. (2012).	Excluded	Study area difference	https://doi.org/10.1002/ajmg.a.34383
59.	Aguilar-Garduño, C., et al. (2010).	Excluded	Study area difference	https://doi.org/10.1136/oem.2008.044743
60.	Ahmed, K. Y., et al. (2020).	Excluded	Unrelated topic	https://doi.org/10.1186/s12889-020-09345-6
61.	Ahrens, K., et al. (2011).	Excluded	Study area difference	https://doi.org/10.1097/ede.0b013e3182227887
62.	Aiemjoy, K., et al. (2019).	Excluded	Unrelated topic	https://doi.org/10.1186/s12879-019-3674-3
63.	Airede, K. I. (1992).	Excluded	Study area difference	https://doi.org/10.1093/tropej/38.1.27
64.	Akar, N., et al. (1988).	Excluded	Study area difference	https://doi.org/10.1111/j.1365-3016.1988.tb00181.x
65.	Aksenov, L. I., et al. (2022).	Excluded	Study area difference	https://doi.org/10.1016/j.jpurol.2022.03.002
66.	Al Rakaf, M. S., et al. (2015).	Excluded	Study area difference	https://doi.org/10.1016/j.pmedr.2015.06.016

67.	Al-Ani, Z. R., et al. (2010).	Excluded	Study area difference	https://pubmed.ncbi.nlm.nih.gov/20174732/
68.	Alasfoor, D., et al. (2010).	Excluded	Study area difference	https://pubmed.ncbi.nlm.nih.gov/20799554/
69.	Alatise, O. I., et al. (2006).	Excluded	Study area difference	https://doi.org/10.1159/000094062
70.	Alembik, Y., et al. (1995).	Excluded	Study area difference	https://pubmed.ncbi.nlm.nih.gov/7625759/
71.	Alemu, A., et al. (2020).	Excluded	Unrelated topic	https://doi.org/10.1186/s12879-020-4817-2
72.	Allagh, K. P., et al. (2015).	Excluded	Study area difference	https://doi.org/10.1371/journal.pone.0118961
73.	Alnaami, I. M. and E. G. Alayad (2019).	Excluded	Study area difference	https://doi.org/10.17712/nsj.2019.1.20180169
74.	Alriksson-Schmidt, A. I., et al. (2021).	Excluded	Study area difference	https://doi.org/10.3233/prm-190661
75.	AlShail, E., et al. (2014).	Excluded	Study area difference	https://pubmed.ncbi.nlm.nih.gov/25551116/
76.	Altan, E., et al. (2018).	Excluded	Unrelated topic	https://doi.org/10.1371/journal.pone.0202054
77.	Alwan, S., et al. (2007).	Excluded	Study area difference	https://doi.org/10.1056/nejmoa066584
78.	Amarin, Z. O. and A. Z. Obeidat (2010).	Excluded	Study area difference	https://doi.org/10.1111/j.1365-3016.2010.01123.x
79.	Amini, H., et al. (2009). "The	Excluded	Study area difference	https://doi.org/10.1080/00016340902934696
80.	Amogne, M. D., et al. (2021).	Excluded	Unrelated topic	https://doi.org/10.1111/j.1365-3016.2010.01123.x
81.	Amouzou, A., et al. (2022).	Excluded	Unrelated topic	https://doi.org/10.1136/bmjgh-2021-008069
82.	Amsalu, E. T., et al. (2019).	Excluded	Unrelated topic	https://doi.org/10.1371/journal.pone.0215572
83.	An, H., et al. (2022).	Excluded	Study area difference	https://doi.org/10.1136/bmjopen-2021-058068
84.	Anane-Fenin, B., et al. (2023).	Excluded	Study area difference	https://doi.org/10.1007/s10995-023-03591-x
85.	Anderka, M., et al. (2012).	Excluded	Study area difference	https://doi.org/10.1002/bdra.22865
86.	Anderson, H. A., et al. (2012).	Excluded	Study area difference	https://doi.org/10.3928/01913913-20120501-01
87.	Aqrabawi, H. E. (2005).	Excluded	Study area difference	https://pubmed.ncbi.nlm.nih.gov/16700398/
88.	Arata, M., et al. (2000).	Excluded	Study area difference	https://pubmed.ncbi.nlm.nih.gov/11027084/
89.	Argaw, M. D., et al. (2021).	Excluded	Unrelated topic	https://doi.org/10.9745/ghsp-d-20-00114
90.	Arth, A., et al. (2015).	Excluded	Study area difference	https://pubmed.ncbi.nlm.nih.gov/25590679/
91.	Arth, A., et al. (2016).	Excluded	Study area difference	https://doi.org/10.1002/bdra.23529
92.	Asres, G., et al. (2011).	Excluded	Unrelated topic	https://doi.org/10.1089/bfm.2011.0016
93.	Assefa, A., et al. (2018).	Excluded	Unrelated topic	https://doi.org/10.1186/s12936-018-2538-4
94.	Assefa, A., et al. (2019).	Excluded	Unrelated topic	https://doi.org/10.1186/s12936-019-2874-z
95.	Assefa, A., et al. (2020).	Excluded	Unrelated topic	https://doi.org/10.1186/s12936-020-03177-w
96.	Astatke, H. and R. Serpell (2000).	Excluded	Unrelated topic	https://doi.org/10.1093/jpepsy/25.6.367
97.	Attenello, F. J., et al. (2016).	Excluded	Study area difference	https://doi.org/10.1007/s00381-016-3165-4
98.	Aubuchon-Endsley, et al. (2013).	Excluded	Unrelated topic	https://doi.org/10.1111/j.1740-8709.2011.00391.x
99.	Auffret, M., et al. (2019).	Excluded	Study area difference	https://doi.org/10.1111/1471-0528.15800

100.	Auger, N., et al. (2019).	Excluded	Study area difference	https://doi.org/10.1080/09638288.2018.1425920
101.	Avagliano, L., et al. (2019).	Excluded	Study area difference	https://doi.org/10.1002/bdr2.1380
102.	Ayaz, R. and M. R. Asoglu (2020).	Excluded	Study area difference	https://doi.org/10.1080/14767058.2019.1623778
103.	Aychiluhm, S. B., et al. (2021).	Excluded	Unrelated topic	https://doi.org/10.1155/2021/1969721
104.	Ayele, W., et al. (2012).	Excluded	Unrelated topic	https://doi.org/10.1093/infdis/jis531
105.	Ayenew, Z., et al. (2021).	Excluded	Unrelated topic	https://doi.org/10.1371/journal.pone.0250896
106.	Aylett, M. J., et al. (1974).	Excluded	Study area difference	https://doi.org/10.1136/jech.28.3.177
107.	Azage, M., et al. (2015).	Excluded	Unrelated topic	https://doi.org/10.1371/journal.pone.0144690
108.	Azage, M., et al. (2017).	Excluded	Unrelated topic	https://doi.org/10.1371/journal.pone.0186933
109.	Ba, G., et al. (2017).	Excluded	Study area difference	https://doi.org/10.18632/oncotarget.14848
110.	Babgi, M. A., et al. (2019).	Excluded	Study area difference	https://doi.org/10.3233/npm-17165
111.	Bachewe, F., et al. (2023).	Excluded	Unrelated topic	https://doi.org/10.1177/03795721231188913
112.	Bakhireva, L. N., et al. (2004).	Excluded	Unrelated topic	https://doi.org/10.7205/milmed.169.3.221
113.	Bamforth, S. J., et al. (1989).	Excluded	Study area difference	https://pubmed.ncbi.nlm.nih.gov/2643314/
114.	Bankole, O. B., et al. (2012).	Excluded	Study area difference	https://pubmed.ncbi.nlm.nih.gov/23175875/
115.	Baradaran, N., et al. (2008).	Excluded	Study area difference	https://doi.org/10.1159/000149900
116.	Baradaran, N., et al. (2009).	Excluded	Study area difference	https://doi.org/10.1159/000277622
117.	Barboza A.M., et al. (2011).	Excluded	Study area difference	https://pubmed.ncbi.nlm.nih.gov/22159644/
118.	Barboza-AML., et al. (2015).	Excluded	Study area difference	https://doi.org/10.1007/s10995-014-1542-8
119.	Barlow-Mosha, L., et al. (2022).	Excluded	Study area difference	https://doi.org/10.1002/bdr2.1964
120.	Bateman, B. T., et al. (2021).	Excluded	Study area difference	https://doi.org/10.1136/bmj.n102
121.	Bean, L. J., et al. (2011).	Excluded	Study area difference	https://doi.org/10.1002/bdra.22848
122.	Beaudin, A. E., et al. (2007).	Excluded	Study area difference	https://doi.org/10.1002/bdrc.20100
123.	Beaumont, M., et al. (2019).	Excluded	Study area difference	https://doi.org/10.1007/s00439-019-01993-y
124.	Behrooz, A. et al. (2007).	Included	Study area difference	https://pubmed.ncbi.nlm.nih.gov/21654942/
125.	Behunova, J., et al. (2010).	Excluded	Study area difference	https://doi.org/10.1002/bdra.20692
126.	Bekele, A., et al. (2022).	Excluded	Unrelated topic	https://doi.org/10.1186/s12887-022-03784-3
127.	Belavady, B. (1978).	Excluded	Unrelated topic	https://doi.org/10.1111/j.1651-2227.1978.tb17803.x
128.	Belay, A., et al. (2020).	Excluded	Unrelated topic	https://doi.org/10.3390/nu12061565
129.	Belay, W., et al. (2022).	Excluded	Unrelated topic	https://doi.org/10.1186/s12872-022-02473-4
130.	Belay, Y. B., et al. (2021).	Excluded	Unrelated topic	https://doi.org/10.1186/s12955-021-01670-7
131.	Belew, M., et al. (2000).	Excluded	Unrelated topic	https://pubmed.ncbi.nlm.nih.gov/11144876/
132.	Belmaker, R. H. (2012).	Excluded	Unrelated topic	https://pubmed.ncbi.nlm.nih.gov/23314088/
133.	Benedum, C. M., et al. (2015).	Excluded	Study area difference	https://doi.org/10.1093/aje/kwv126

134.	Benjamin, R. H., et al. (2019).	Excluded	Study area difference	https://doi.org/10.1002/bdr2.1547
135.	Berhanu, D., et al. (2020).	Excluded	Unrelated topic	https://doi.org/10.1186/s12913-020-05151-3
136.	Berhanu, D., et al. (2021).	Excluded	Unrelated topic	https://doi.org/10.1371/journal.pone.0251706
137.	Berhe, R. and A. Nigusie (2020).	Excluded	Unrelated topic	https://doi.org/10.1186/s12889-020-08919-8
138.	Berry, R. J., et al. (1999).	Excluded	Study area difference	https://doi.org/10.1056/nejm199911113412001
139.	Best, K. E., et al. (2018).	Excluded	Study area difference	https://doi.org/10.1016/j.ejmg.2018.05.012
140.	Beyera, G. K., et al. (2020)	Excluded	Unrelated topic	https://doi.org/10.1111/1756-185x.13832
141.	Bhandari, S., et al. (2015).	Excluded	Study area difference	https://doi.org/10.1186/s12887-015-0453-1
142.	Bhide, P. and A. Kar (2018).	Excluded	Study area difference	https://doi.org/10.1186/s12887-018-1149-0
143.	Bhide, P., et al. (2013).	Excluded	Study area difference	https://doi.org/10.1002/bdra.23153
144.	Bhide, P., et al. (2016).	Excluded	Study area difference	https://doi.org/10.1371/journal.pone.0166408
145.	Biadgilign, S., et al. (2023)	Excluded	Unrelated topic	https://doi.org/10.1186/s12992-023-00949-2
146.	Biadglegne, F., et al. (2014)	Excluded	Unrelated topic	https://doi.org/10.1371/journal.pone.0106869
147.	Bidondo, M. P., et al. (2016)	Excluded	Study area difference	https://doi.org/10.1002/bdra.23549
148.	Biluts, H.,et al. (2016)	Excluded	Unrelated topic	https://doi.org/10.1016/j.wneu.2016.04.114
149.	Birnbacher, R., et al. (2002).	Excluded	Study area difference	https://doi.org/10.1097/00042307-200211000-00002
150.	Bitsko, R. H., et al. (2007).	Excluded	Study area difference	https://doi.org/10.1002/ajmg.a.31950
151.	Bittar, Z. (1998).	Excluded	Study area difference	https://pubmed.ncbi.nlm.nih.gov/10349259/
152.	Blaisdell, J., et al. (2019)	Excluded	Study area difference	https://doi.org/10.1016/j.envres.2019.108553
153.	Blanco Muñoz, J., et al. (2005).	Excluded	Study area difference	https://doi.org/10.1177/003335490512000108
154.	Blanton, S. H., et al. (2011).	Excluded	Study area difference	https://doi.org/10.1002/bdra.20740
155.	Blatter, B. M., et al. (1997)	Excluded	Study area difference	https://doi.org/10.1002/(sici)1096-9926(199704
156.	Blencowe, H., et al. (2018).	Excluded	Study area difference	https://doi.org/10.1111/nyas.13548
157.	Blomberg, M. I.,et al. (2010).	Excluded	Study area difference	https://doi.org/10.1002/bdra.20620
158.	Blount, J. P., et al. (2021).	Excluded	Study area difference	https://doi.org/10.4103/0028-3886.332247
159.	Bochud, P. Y., et al. (2008)	Excluded	Unrelated topic	https://doi.org/10.1086/524688
160.	Boduroğlu, K., et al. (2005).	Excluded	Study area difference	https://pubmed.ncbi.nlm.nih.gov/16363341/
161.	Bol, K. A., et al. (2006).	Excluded	Study area difference	https://doi.org/10.1542/peds.2005-1364
162.	Bolme, P., et al. (1995).	Excluded	Unrelated topic	https://doi.org/10.1111/j.1600-0773.1995.tb00140.x
163.	Boo, N. Y., et al. (2013).	Excluded	Study area difference	https://doi.org/10.1093/tropej/fmt026
164.	Borelli, M., et al. (2017).	Excluded	Topic difference	https://doi.org/10.1002/ajmg.a.38013
165.	Borgstedt-Bakke., et al. (2017).	Excluded	Study area difference	https://doi.org/10.3171/2016.8.peds1654
166.	Borman, B. and C. Cryer (1993).	Excluded	Study area difference	https://doi.org/10.1111/j.1440-1754.1993.tb00512.x
167.	Borman, G. B., et al. (1986).	Excluded	Study area difference	https://doi.org/10.1002/tera.1420330209

168.	Botto, L. D., et al. (2005).	Excluded	Study area difference	https://doi.org/10.1136/bmj.38336.664352.82
169.	Boulet, S. L., et al. (2008).	Excluded	Study area difference	https://doi.org/10.1002/bdra.20468
170.	Boulton, M. L., et al. (2019).	Excluded	Unrelated topic	https://doi.org/10.1371/journal.pone.0212408
171.	Bower, C. and F. J. Stanley (2004).	Excluded	Study area difference	https://doi.org/10.1002/bdra.20074
172.	Bower, C., et al. (1984).	Excluded	Study area difference	https://doi.org/10.1136/jech.38.3.208
173.	Bower, C., et al. (1993)	Excluded	Study area difference	https://doi.org/10.5694/j.1326-5377.1993.tb121690.x
174.	Bower, C., et al. (2002).	Excluded	Study area difference	https://doi.org/10.1111/j.1467-842x.2002.tb00908.x
175.	Bower, C., et al. (2004).	Excluded	Study area difference	https://doi.org/10.1111/j.1365-3016.2004.00567.x
176.	Bowkett, B. and E. Deveral (2012).	Excluded	Study area difference	https://pubmed.ncbi.nlm.nih.gov/22426607/
177.	Bowman, R. M., et al. (2009).	Excluded	Study area difference	https://doi.org/10.1007/s00381-009-0865-z
178.	Bowman, R. M., et al. (2009).	Excluded	Study area difference	https://doi.org/10.1007/s00381-009-0865-z
179.	Bowser, D., et al. (2023).	Excluded	Unrelated topic	https://doi.org/10.1371/journal.pone.0291958
180.	Boyd, P. A., et al. (2000).	Excluded	Study area difference	https://doi.org/10.1136/jms.7.4.169
181.	Brekke, I., et al. (2017)	Excluded	Study area difference	https://doi.org/10.1186/s12887-016-0774-8
182.	Bremer, S., et al. (2018).	Excluded	Study area difference	https://doi.org/10.1055/s-0042-114082.
183.	Brender, J. D. and L. Suarez (1990).	Excluded	Study area difference	https://doi.org/10.1093/oxfordjournals.aje.a115526
184.	Brender, J. D., et al. (2008)	Excluded	Study area difference	https://pubmed.ncbi.nlm.nih.gov/18507277/
185.	Brender, J. D., et al. (2014).	Excluded	Study area difference	https://doi.org/10.1186/1476-069x-13-96
186.	Bronberg, R., et al. (2011).	Excluded	Study area difference	https://www.academia.edu/14554853
187.	Bronberg, R., et al. (2020)	Excluded	Study area difference	https://doi.org/10.1007/s12687-019-00449-0
188.	Bronberg, R., et al. (2023).	Excluded	Study area difference	https://doi.org/10.1002/bdr2.2248
189.	Brosnan, J. T., et al. (2018).	Excluded	Study area difference	https://doi.org/10.1093/ajcn/nqx065
190.	Brownlee, E. M., et al. (2009).	Excluded	Study area difference	https://doi.org/10.1016/j.jpedsurg.2008.10.076
191.	Buccimazza, S. S., et al. (1994).	Excluded	Study area difference	https://doi.org/10.1002/tera.1420500304
192.	Budge, S., et al. (2021).	Excluded	Unrelated topic	https://doi.org/10.4269/ajtmh.20-0945
193.	Bupp, C. P., et al. (2015).	Excluded	Study area difference	https://doi.org/10.1002/ajmg.a.37168
194.	Byrne, J. (2008).	Excluded	Study area difference	https://doi.org/10.1002/bdra.20406
195.	Byrne, J. (2010).	Excluded	Study area difference	https://doi.org/10.1007/s11845-009-0381-x
196.	Cai, C. Q., et al. (2019).	Excluded	Study area difference	https://doi.org/10.1186/s13052-019-0630-1
197.	Calzolari, E., et al. (2004).	Excluded	Study area difference	https://doi.org/10.1597/02-074.1
198.	Cameron, C. M., et al. (2014).	Excluded	Study area difference	https://doi.org/10.1038/ijo.2014.148
199.	Campagna, A. M., et al. (2013).	Excluded	Unrelated topic	https://doi.org/10.1016/j.mayocp.2012.07.027
200.	Canfield, M. A., et al. (2006).	Excluded	Study area difference	https://doi.org/10.1038/sj.jes.7500501

201.	Cardona-Grau, D. and G. Chiang (2017).	Excluded	Study area difference	https://doi.org/10.1016/j.ucl.2017.04.006
202.	Carmichael, S. L., et al. (2002)	Excluded	Study area difference	https://doi.org/10.1023/a:1019722011688
203.	Carmichael, S. L., et al. (2014).	Excluded	Study area difference	https://doi.org/10.1111/ppe.12123
204.	Carter, T. C., et al. (2008).	Excluded	Study area difference	https://doi.org/10.1016/j.ajog.2007.08.044
205.	Case, A. P., et al. (2007).	Excluded	Study area difference	https://doi.org/10.1111/j.1552-6909.2007.00163.x
206.	Cate, I. M., et al. (2002).	Excluded	Study area difference	https://doi.org/10.1017/s0012162201002146
207.	Cavalari, K. N., et al. (2017).	Excluded	Study area difference	https://doi.org/10.1016/j.pedn.2017.07.005
208.	Cavalcanti, D. P. and M. A. Salomão (2003).	Excluded	Study area difference	https://pubmed.ncbi.nlm.nih.gov/14502334/
209.	Çaylan, N., et al. (2022).	Excluded	Study area difference	https://doi.org/10.1186/s12884-022-04678-z
210.	Celentano, I., et al. (2021).	Excluded	Unrelated topic	https://doi.org/10.1186/s12889-021-11005-2
211.	Chan, A. C., et al. (2008).	Excluded	Study area difference	https://doi.org/10.5694/j.1326-5377.2008.tb02183.x
212.	Chanarin, I. (1994).	Excluded	Study area difference	https://pubmed.ncbi.nlm.nih.gov/7924000/
213.	Che, X., et al. (2022).	Excluded	Study area difference	https://doi.org/10.3390/biology11091371
214.	Chekesa, B., et al. (2020).	Excluded	Unrelated topic	https://doi.org/10.1371/journal.pone.0233314
215.	Chekol, Y. M., et al. (2023).	Excluded	Unrelated topic	https://doi.org/10.1371/journal.pone.0288710
216.	Chelkeba, L., et al. (2020).	Excluded	Unrelated topic	https://doi.org/10.1186/s12889-020-8222-y
217.	Chen, B. H., et al. (2008).	Excluded	Study area difference	https://doi.org/10.1002/bdra.20466
218.	Chen, G., et al. (2009).	Excluded	Study area difference	https://doi.org/10.1016/s0895-3988(09)60065-9
219.	Chen, L. T. and M. A. Rivera (2004).	Excluded	Study area difference	https://doi.org/10.1111/j.1753-4887.2004.tb00073.x
220.	Cheng, N., et al. (2003).	Excluded	Study area difference	https://doi.org/10.1179/000349803125002823
221.	Cherian, A. G., et al. (2016).	Included	Study area difference	https://doi.org/10.1093/tropej/fmw019
222.	Cherian, A., et al. (2005).	Excluded	Study area difference	https://doi.org/10.1016/s0140-6736(05)67319-9
223.	Cherian, J., et al. (2016).	Excluded	Study area difference	https://doi.org/10.3171/2016.1.peds15674
224.	Childebayeva, A., et al. (2019).	Excluded	Unrelated topic	https://doi.org/10.1080/15592294.2018.1561117
225.	Chu, H., et al. (2021).	Excluded	Unrelated topic	https://doi.org/10.1016/j.vaccine.2021.05.029
226.	Clarke, C., et al. (1975).	Excluded	Study area difference	https://doi.org/10.1136/bmj.4.5999.743
227.	Claude, K. M., et al. (2012).	Excluded	Study area difference	https://doi.org/10.1111/j.1740-8709.2010.00271.x
228.	Coard, K., et al. (1990).	Excluded	Study area difference	https://doi.org/10.1002/tera.1420410208
229.	Colapinto, C. K., et al. (2011).	Excluded	Study area difference	https://doi.org/10.1503/cmaj.100568
230.	Coleman, B. G., et al. (2015).	Excluded	Study area difference	https://doi.org/10.1159/000364806
231.	Collins, J. S., et al. (2009).	Excluded	Study area difference	https://doi.org/10.1002/bdra.20619
232.	Collins, J. S., et al. (2011)	Excluded	Study area difference	https://doi.org/10.1016/j.jpeds.2010.12.037

233.	Cordero, A. M., et al. (2015).	Excluded	Study area difference	https://pubmed.ncbi.nlm.nih.gov/25905896/
234.	Cordier, S., et al. (1997).	Excluded	Study area difference	https://doi.org/10.1097/00001648-199707000-00002
235.	Cornell, J., et al. (1983).	Included	Study area difference	https://pubmed.ncbi.nlm.nih.gov/6346521/
236.	Corroenne, R., et al. (2020).	Excluded	Study area difference	https://doi.org/10.1002/uog.21947
237.	Costa, C. M., et al. (2006).	Excluded	Study area difference	https://doi.org/10.1590/s0102-311x2006001100016
238.	Costenbader, E., et al. (2020)	Excluded	Unrelated topic	https://doi.org/10.9745/ghsp-d-20-00135
239.	Cowan, J., et al. (2013).	Excluded	Unrelated topic	https://doi.org/10.5588/ijtld.12.0240
240.	Crandall, B. F., et al. (1983).	Excluded	Study area difference	https://pubmed.ncbi.nlm.nih.gov/6191442/
241.	Crider, K. S., et al. (2011).	Excluded	Study area difference	https://doi.org/10.3390/nu3030370
242.	Crider, K. S., et al. (2018).	Excluded	Study area difference	https://doi.org/10.1093/ajcn/nqy065
243.	Croen, L. A., et al. (2001).	Excluded	Study area difference	https://doi.org/10.1093/aje/153.4.325
244.	Crytzer, T. M., et al. (2013).	Excluded	Study area difference	https://doi.org/10.1016/j.pmrj.2013.06.010
245.	Csermely, G., et al. (2014).	Excluded	Study area difference	https://doi.org/10.1016/j.ejogrb.2014.05.035
246.	Csermely, G., et al. (2015).	Excluded	Study area difference	https://doi.org/10.3109/14767058.2014.918946
247.	Cuckle, H. and N. Wald (1987).	Excluded	Study area difference	https://doi.org/10.1002/pd.1970070205
248.	Curry, L. A., et al. (2013)	Excluded	Unrelated topic	https://doi.org/10.1371/journal.pone.0079847
249.	Czapran, P., et al. (2012)	Excluded	Study area difference	https://doi.org/10.3109/02688697.2011.635822
250.	Czeizel, A. and G. Karig (1985).	Excluded	Study area difference	https://pubmed.ncbi.nlm.nih.gov/3939087/
251.	Czeizel, A. E. (2004).	Excluded	Study area difference	https://doi.org/10.7150/ijms.1.50
252.	Da Silva, S. L., et al. (2015).	Excluded	Study area difference	https://doi.org/10.3171/2014.10.peds14192
253.	Dalal, A., et al. (2007).	Excluded	Study area difference	https://doi.org/10.1159/000096735
254.	Damtie, D., et al. (2023).	Excluded	Unrelated topic	https://doi.org/10.1371/journal.pone.0295170
255.	de Franchis, R., et al. (1998)	Excluded	Study area difference	https://doi.org/10.1136/jmg.35.12.1009
256.	De Groote, H., et al. (2021)	Excluded	Unrelated topic	https://doi.org/10.1038/s41598-021-88304-6
257.	de Jong, T. P., et al. (2008).	Excluded	Study area difference	https://doi.org/10.1007/s00467-008-0780-7
258.	De Marco, P., et al. (2011).	Excluded	Study area difference	https://doi.org/10.1007/s00381-010-1372-y
259.	de Paul Djientcheu, V., et al. (2008).	Excluded	Study area difference	https://doi.org/10.1007/bf03404123
260.	De Wals, P., et al. (1999).	Excluded	Study area difference	https://doi.org/10.1007/bf03404123
261.	Deak, K. L., et al. (2008).	Excluded	Study area difference	https://doi.org/10.1002/bdra.20511
262.	Dean, J. H., et al. (2020).	Excluded	Study area difference	
263.	Deb, R., et al. (2014).	Excluded	Study area difference	https://doi.org/10.1017/s1368980012005393
264.	Deb, R., et al. (2017).	Excluded	Study area difference	https://doi.org/10.1002/bdr2.1126
265.	Deb, R., et al. (2018).	Excluded	Study area difference	https://doi.org/10.1002/bdr2.1373
266.	Defar, A., et al. (2021).	Excluded	Unrelated topic	https://doi.org/10.3390/ijerph18073816

267.	Degarege, A., et al. (2016).	Excluded	Unrelated topic	https://doi.org/10.1371/journal.pntd.0005193
268.	Dejene, T. and E. Gurmu (2022).	Excluded	Unrelated topic	https://doi.org/10.1371/journal.pone.0279773
269.	Deroche, C. B., et al. (2015).	Excluded	Study area difference	https://doi.org/10.1016/j.ridd.2014.12.009
270.	Deshmukh, S. S. (1997).	Excluded	Study area difference	https://pubmed.ncbi.nlm.nih.gov/11129882/
271.	Desrosiers, T. A., et al. (2012)	Excluded	Study area difference	https://doi.org/10.1136/oemed-2011-100245
272.	Desrosiers, T. A., et al. (2018).	Excluded	Study area difference	https://doi.org/10.1002/bdr2.1198
273.	Dessie, Z. B., et al. (2019)	Excluded	Unrelated topic	https://doi.org/10.1186/s12887-019-1459-x
274.	Detrait, E. R., et al. (2005)	Excluded	Study area difference	https://doi.org/10.1016/j.ntt.2004.12.007
275.	Dhiman, S., et al. (2020).	Excluded	Study area difference	https://doi.org/10.1016/j.ridd.2020.103790
276.	Dillon, C. M., et al. (2000)	Excluded	Study area difference	https://doi.org/10.1186/s12887-019-1459-x
277.	Dinede, G., et al. (2019)	Excluded	Unrelated topic	https://doi.org/10.1016/j.ntt.2004.12.007
278.	Diriba, G., et al. (2020)	Excluded	Unrelated topic	https://doi.org/10.1016/j.ridd.2020.103790
279.	du Plessis, L., et al. (2008)	Excluded	Study area difference	https://doi.org/10.5694/j.1326-5377.2008.tb02184.x
280.	Dupépé, E. B., et al. (2017).	Excluded	Study area difference	https://doi.org/10.3171/2016.12.peds1668
281.	Eaves, L. A., et al. (2023).	Excluded	Study area difference	https://doi.org/10.1289/ehp11872
282.	Ebrahim, N. B., et al. (2016)	Excluded	Unrelated topic	https://doi.org/10.1111/phn.12281
283.	Ejigu, B. A., et al. (2018)	Excluded	Unrelated topic	https://doi.org/10.1371/journal.pone.0197171
284.	Ekwochi, U., et al. (2018)	Excluded	Study area difference	https://doi.org/10.1093/tropej/fmx067
285.	Eldridge, C., et al. (2018).	Excluded	Study area difference	https://doi.org/10.1002/bdr2.1221
286.	El-Shabrawi, M. H., et al. (2015)	Excluded	Study area difference	https://doi.org/10.1097/md.0000000000001395
287.	Enbiale, W., et al. (2020)	Excluded	Unrelated topic	https://doi.org/10.3855/jidc.11701
288.	Enden, M. R., et al. (2021)	Excluded	Unrelated topic	https://doi.org/10.1016/j.socscimed.2021.114076
289.	Engidaye, G., et al. (2019)	Excluded	Unrelated topic	https://doi.org/10.1186/s12889-019-7293-0
290.	Espira, L. M., et al. (2023)	Excluded	Unrelated topic	https://doi.org/10.1021/acs.est.2c08976
291.	Esposito, D. B., et al. (2021).	Excluded	Study area difference	https://doi.org/10.1002/bdr2.1944
292.	Estevez-Ordonez, D., et al. (2018).	Excluded	Study area difference	https://doi.org/10.3171/2018.7.focus18231
293.	Etheredge, A. J., et al. (2012)	Excluded	Study area difference	https://doi.org/10.1002/ajmg.a.35552
294.	Fantahun, M., et al. (2019)	Excluded	Unrelated topic	https://doi.org/10.1371/journal.pone.0222402
295.	Fantay Gebru, K., et al. (2019)	Excluded	Unrelated topic	https://doi.org/10.1186/s12887-019-1545-0
296.	Fawibe, A. E., et al. (2017)	Excluded	Unrelated topic	https://doi.org/10.1186/s12890-017-0390-x
297.	Fear, N. T., et al. (2007)	Excluded	Study area difference	https://doi.org/10.1111/j.1365-3016.2007.00793.x
298.	Fekadu, D., et al. (2006)	Excluded	Unrelated topic	https://doi.org/10.1111/j.1469-7610.2006.01617.x
299.	Feldkamp, M., et al. (2002).	Excluded		https://doi.org/10.1002/tera.90006
300.	Feldman, J. G., et al. (1982)	Excluded	Study area difference	https://doi.org/10.1016/0021-9681(82)90029-7
301.	Feleke, S. M., et al. (2021)	Excluded	Unrelated topic	https://doi.org/10.1038/s41564-021-00962-4

302.	Felkner, M., et al. (2003).	Excluded	Study area difference	https://doi.org/10.1002/bdra.10047
303.	Fentie, A. M., et al. (2022)	Excluded	Unrelated topic	https://doi.org/10.1136/bmjopen-2021-054541
304.	Feyisa, G. C., et al. (2023)	Excluded	Unrelated topic	https://doi.org/10.1186/s12889-023-17356-2
305.	Fikre, A., et al. (2011)	Excluded	Unrelated topic	https://doi.org/10.1016/j.fct.2010.09.035
306.	Finkelstein, J. L., et al. (2021).	Excluded	Study area difference	https://doi.org/10.1093/cdn/nzab069
307.	Finkelstein, J. L., et al. (2023)	Excluded	Study area difference	https://doi.org/10.1016/j.cdnut.2023.100052
308.	Finnell, R. H., et al. (2002).	Excluded	Study area difference	https://doi.org/10.1089/109065702760093915
309.	Finnell, R. H., et al. (2021)	Excluded	Study area difference	https://doi.org/10.3389/fgene.2021.659612
310.	Fisher, S. C., et al. (2018).	Excluded	Study area difference	https://doi.org/10.1002/bdr2.1372
311.	Flak, A. L., et al. (2012).	Excluded	Study area difference	https://doi.org/10.1002/bdra.23017
312.	Flood, L., et al. (2013)	Excluded	Study area difference	https://doi.org/10.5365/wpsar.2012.3.3.006
313.	Flores, A. L., et al. (2014).	Excluded	Study area difference	https://pubmed.ncbi.nlm.nih.gov/26120254/
314.	Forci, K., et al. (2021).	Excluded	Study area difference	https://doi.org/10.1186/s12887-021-02584-5
315.	Ford, N., et al. (2014).	Excluded	Study area difference	https://doi.org/10.1097/qad.0000000000000231
316.	Forrester, M. B. and R. D. Merz (2004).	Excluded	Study area difference	https://doi.org/10.1002/bdra.20092
317.	Forrester, M. B and R. D. Merz (2008).	Excluded	Study area difference	https://doi.org/10.1111/j.1741-4520.2008.00184.x
318.	Franke, B., et al. (2009)	Excluded	Study area difference	https://doi.org/10.1002/bdra.20556
319.	Frey, L. and W. A. Hauser (2003).	Excluded	Study area difference	https://doi.org/10.1046/j.1528-1157.44.s3.2.x
320.	Fujimori, E., et al. (2013).	Excluded	Study area difference	https://doi.org/10.1590/S0102-311X2013000100017
321.	Gage, A. D., et al. (2018)	Excluded	Unrelated topic	https://doi.org/10.1371/journal.pone.0208898
322.	Gao, L. J., et al. (2013).	Excluded	Study area difference	https://doi.org/10.1002/bdra.23123
323.	García-Fragoso, L., et al. (2008)	Excluded	Study area difference	https://pmc.ncbi.nlm.nih.gov/articles/PMC2747812/
324.	Garne, E., et al. (2012).	Excluded	Study area difference	https://doi.org/10.1002/bdra.22886
325.	Gashu, D., et al. (2016)	Excluded	Unrelated topic	https://doi.org/10.1017/s1368980015003626
326.	Gass, K. M., et al. (2017)	Excluded	Unrelated topic	https://doi.org/10.1371/journal.pntd.0005944
327.	Gebre, B., et al. (2018)	Excluded	Unrelated topic	https://doi.org/10.1093/tropej/fmx031
328.	Gebre-Medhin, M. and A. Vahlquist (1984).	Excluded	Unrelated topic	https://doi.org/10.1111/j.1651-2227.1994.tb17744.x
329.	Gebre-Medhin, M. and G. Birgegård (1981).	Excluded	Unrelated topic	https://doi.org/10.1186/1471-2326-9-7
330.	Gebremedhin, T. A. and I. Mohanty (2016)	Excluded	Unrelated topic	https://doi.org/10.1371/journal.pone.0167639
331.	Gedefaw, G., et al. (2020)	Excluded	Unrelated topic	https://doi.org/10.1371/journal.pone.0244229

332.	Geibel, S., et al. (2016)	Excluded	Unrelated topic	https://doi.org/10.1371/journal.pone.0147267
333.	Gejo, N. G., et al. (2019)	Excluded	Unrelated topic	https://doi.org/10.1371/journal.pone.0210782
334.	Gelaw, N. B., et al. (2022)	Excluded	Unrelated topic	https://doi.org/10.1371/journal.pone.0277565
335.	Gelaye, B., et al. (2013)	Excluded	Unrelated topic	https://doi.org/10.1111/j.1526-4610.2012.02259.x
336.	Gelaye, K. A., et al. (2018)	Excluded	Unrelated topic	https://doi.org/10.1080/16549716.2018.1430669
337.	Gelineau-van Waes, J., et al. (2009).	Excluded	Study area difference	https://doi.org/10.1016/s1043-4526(08)00605-0
338.	Germossa, G. N., et al. (2022)	Excluded	Unrelated topic	https://doi.org/10.1186/s12913-022-08712-w
339.	Gernand, A. D., et al. (2016).	Excluded	Study area difference	https://doi.org/10.1038/nrendo.2016.37
340.	Getachew, B., et al. (2020)	Excluded	Unrelated topic	https://doi.org/10.1371/journal.pone.0224220
341.	Getahun, M., et al. (2016)	Excluded	Unrelated topic	https://doi.org/10.1186/s12879-016-1457-7
342.	Getahun, M., et al. (2017)	Excluded	Unrelated topic	https://doi.org/10.1186/s12879-017-2183-5
343.	Getaneh, Y., et al. (2020)	Excluded	Unrelated topic	https://doi.org/10.1016/j.ijid.2020.02.031
344.	Getaneh, Y., et al. (2023)	Excluded	Unrelated topic	https://doi.org/10.1080/07853890.2023.2239828
345.	Geweniger, A. and K. M. Abbas (2020)	Excluded	Unrelated topic	https://doi.org/10.1016/j.vaccine.2020.03.040
346.	Gezie, L. D., et al. (2019)	Excluded	Unrelated topic	https://doi.org/10.1136/bmjopen-2018-024515
347.	Ghalib, H. W., et al. (1987)	Excluded	Unrelated topic	https://doi.org/10.1080/00034983.1987.11812138
348.	Ghosh, S., et al. (2019)	Excluded	Unrelated topic	https://doi.org/10.1371/journal.pone.0214358
349.	Ghotme, K. A., et al. (2023).	Excluded	Study area difference	https://doi.org/10.1007/s00381-023-05944-x
350.	Gibson, M. A., et al. (2018)	Excluded	Unrelated topic	https://doi.org/10.1371/journal.pone.0193985
351.	Gidey, A. B., et al. (2021)	Excluded	Unrelated topic	https://doi.org/10.1371/journal.pone.0260104
352.	Gilbertson, K. E., et al. (2023).	Excluded	Study area difference	https://doi.org/10.1097/dbp.0000000000001218
353.	Gildestad, T., et al. (2020).	Excluded	Study area difference	https://doi.org/10.1017/s0007114520001178
354.	Gill, M. M., et al. (2023).	Excluded	Study area difference	https://doi.org/10.1093/ofid/ofad441
355.	Girguis, M. S., et al. (2016).	Excluded	Study area difference	https://doi.org/10.1016/j.envres.2015.12.010
356.	Girma, T., et al. (2022)	Excluded	Unrelated topic	https://doi.org/10.1371/journal.pone.0264719
357.	Girmay, A. M., et al. (2023)	Excluded	Unrelated topic	https://doi.org/10.1136/bmjopen-2022-071296
358.	Githuku, J. N., et al. (2014).	Excluded	Study area difference	https://doi.org/10.11604/pamj.2014.18.60.4070
359.	Godebo, T. R., et al. (2019)	Excluded	Unrelated topic	https://doi.org/10.1016/j.ijheh.2018.12.007
360.	Golalipour, M. J., et al. (2007).	Excluded	Study area difference	https://pubmed.ncbi.nlm.nih.gov/17687828/
361.	Golalipour, M. J., et al. (2010).	Excluded	Study area difference	https://pubmed.ncbi.nlm.nih.gov/20039767/
362.	Gong, R., et al. (2010)	Excluded	Study area difference	https://doi.org/10.1002/bdra.20691
363.	Graham, A., et al. (2010)	Excluded	Study area difference	https://doi.org/10.1002/bdra.20672
364.	Groisman, B., et al. (2013)	Excluded	Study area difference	https://doi.org/10.1007/s12687-012-0120-2
365.	Groot, J., et al. (2022).	Excluded	Study area difference	https://doi.org/10.1093/ajcn/nqab351

366.	Grosse, S. D. and J. S. Collins (2007)	Excluded	Study area difference	https://doi.org/10.1002/bdra.20394
367.	Grosse, S. D., et al. (2016).	Excluded	Study area difference	https://doi.org/10.1016/j.amepre.2015.10.012
368.	Gu, X., et al. (2007)	Excluded	Study area difference	https://doi.org/10.1002/bdra.20397
369.	Gudu, W. and D. Bekele (2015)	Excluded	Unrelated topic	https://doi.org/10.1186/s13256-015-0712-7
370.	Guéant-Rodriguez, R. M., et al. (2018).	Excluded	Unrelated topic	https://doi.org/10.1111/cge.13104
371.	Gugssa, S. A., et al. (2011)	Excluded	Unrelated topic	https://pubmed.ncbi.nlm.nih.gov/21991757/
372.	Guo, L., et al. (2011)	Excluded	Study area difference	https://pubmed.ncbi.nlm.nih.gov/21362336/
373.	Guthrie, B. L., et al. (2021)	Excluded	Unrelated topic	https://doi.org/10.7189/jogh.11.04063
374.	Gutu, M. A., et al. (2021)	Excluded	Unrelated topic	https://doi.org/10.1371/journal.pntd.0008992
375.	Haase, J., et al. (1987)	Excluded	Study area difference	https://doi.org/10.1007/bf00271138
376.	Habte, D., et al. (2013)	Excluded	Unrelated topic	https://doi.org/10.1371/journal.pone.0082987
377.	Hadley, C., et al. (2011)	Excluded	Unrelated topic	https://doi.org/10.1002/ajpa.21463
378.	Hagaman, A. K., et al. (2020)	Excluded	Unrelated topic	https://doi.org/10.1186/s12913-020-05391-3
379.	Haider, B. A. and Z. A. Bhutta (2017)	Excluded	Study area difference	https://doi.org/10.1002/14651858.cd004905.pub5
380.	Haik, J., et al. (2016)	Excluded	Unrelated topic	https://pubmed.ncbi.nlm.nih.gov/28095560/
381.	Haile, D., et al. (2016)	Excluded	Unrelated topic	https://doi.org/10.1186/s12887-016-0587-9
382.	Haile, D., et al. (2017)	Excluded	Unrelated topic	https://doi.org/10.1186/s12884-016-1210-7
383.	Hailemeskel, S., et al. (2022)	Excluded	Unrelated topic	https://doi.org/10.1016/j.wombi.2021.08.008
384.	Hailu, A. W., et al. (2021)	Excluded	Unrelated topic	https://doi.org/10.1371/journal.pone.0253186
385.	Hailu, C., et al. (2022)	Excluded	Unrelated topic	https://doi.org/10.1186/s12879-022-07350-1
386.	Hakim, W. S. and A. S. Aljanabi (2023)	Excluded	Study area difference	https://doi.org/10.25122/jml-2023-0190
387.	Hall, N. B., et al. (2017)	Excluded	Study area difference	http://dx.doi.org/10.15585/mmwr.mm6631a5
388.	Hamner, H. C., et al. (2011)	Excluded	Unrelated topic	https://doi.org/10.3945/jn.111.143412
389.	Han, J. S., et al. (2023).	Excluded	Study area difference	https://doi.org/10.3171/2023.5.peds22564
390.	Hanlon, C., et al. (2016)	Excluded	Unrelated topic	https://doi.org/10.1111/cch.12371
391.	Harris, D. A., et al. (2016).	Excluded	Study area difference	https://doi.org/10.1007/s00381-016-3091-5
392.	Hasler, M., et al. (2023).	Excluded	Study area difference	https://doi.org/10.1111/cga.12516
393.	Haug, S., et al. (2010)	Excluded	Unrelated topic	https://doi.org/10.1086/655697
394.	Headey, D. and K. Hirvonen (2016)	Excluded	Unrelated topic	https://doi.org/10.1371/journal.pone.0160590
395.	Heck, J. E., et al. (2020)	Excluded	Study area difference	https://doi.org/10.1080/08880018.2020.1760409
396.	Helfrecht, C., et al. (2018)	Excluded	Unrelated topic	https://doi.org/10.1002/ajhb.23090
397.	Hendricks, K. A., et al. (2001).	Excluded	Study area difference	https://doi.org/10.1097/00001648-200111000-00009
398.	Hendriksen, R. S., et al. (2009)	Excluded	Unrelated topic	https://doi.org/10.1097/inf.0b013e3181a3aeac
399.	Herman, A. A. and K. F. Yu (1997).	Excluded	Unrelated topic	https://doi.org/10.1046/j.1365-3016.11.s1.5.x

400.	Hershko, C., et al. (1986)	Excluded	Unrelated topic	https://pubmed.ncbi.nlm.nih.gov/3464761/
401.	Heseker, H. (2011).	Excluded	Study area difference	https://doi.org/10.1159/000332126
402.	Heseker, H. B., et al. (2009)	Excluded	Study area difference	https://doi.org/10.1017/s0007114508149200
403.	Hey, K., et al. (1994)	Excluded	Study area difference	https://doi.org/10.1136/fn.71.3.f198
404.	Hida, K., et al. (1994).	Excluded	Study area difference	https://doi.org/10.1136/jnnp.57.3.373
405.	Higashi, H., et al. (2015)	Excluded	Study area difference	https://doi.org/10.1136/archdischild-2014-306175
406.	Hill, S. M., et al. (2012)	Excluded	Study area difference	https://doi.org/10.1136/fn.71.3.f198
407.	Himmetoglu, O., et al. (1996).	Excluded	Study area difference	https://doi.org/10.1016/s0020-7292(96)02743-9
408.	Ho, P., et al. (2021)	Excluded	Study area difference	https://doi.org/10.1371/journal.pone.0250098
409.	Hoang, T. T., et al. (2018).	Excluded	Study area difference	https://doi.org/10.1002/bdr2.1116
410.	Hoang, T. T., et al. (2019).	Excluded	Study area difference	https://doi.org/10.1093/jn/nxy246
411.	Honein, M. A., et al. (2013)	Excluded	Unrelated topic	https://doi.org/10.1002/oby.20156
412.	Hong, S., et al. (1996)	Excluded	Study area difference	https://doi.org/10.1016/0029-7844(96)00202-5
413.	Howards, P. P., et al. (2015).	Excluded	Study area difference	https://doi.org/10.1093/aje/kwu323
414.	Hoyt, A. T., et al. (2016).	Excluded	Study area difference	https://doi.org/10.1016/j.ajog.2016.07.022
415.	Hrapcak, S., et al. (2021)	Excluded	Unrelated topic	https://doi.org/10.1097/inf.0000000000003324
416.	Huang, J., et al. (2011)	Excluded	Study area difference	https://doi.org/10.3967/0895-3988.2011.02.002
417.	Hunt, G. M. (1983).	Excluded	Study area difference	https://doi.org/10.1055/s-2008-1063090
418.	Hunter, A. G. (1984)	Excluded	Study area difference	https://doi.org/10.1002/ajmg.1320190108
419.	Hunter, A. G., et al. (1996)	Excluded	Study area difference	https://doi.org/10.1002/(sici)1096-9926(199611)54:5%3C213::aid-tera1%3E3.0.co;2-0
420.	Isaacson, G. and A. Melaku (2016)	Excluded	Unrelated topic	https://doi.org/10.1002/lary.25486
421.	Ito, K., et al. (2019).	Excluded	Study area difference	https://doi.org/10.2188/jea.je20170185
422.	Jaikrishan, G., et al. (2013).	Excluded	Study area difference	https://doi.org/10.1007/s12687-012-0113-1
423.	Jain, A., et al. (2018)	Excluded	Unrelated topic	https://doi.org/10.1186/s12978-018-0607-3
424.	James, W. H. (1980)	Excluded	Study area difference	https://doi.org/10.1111/j.1469-8749.1980.tb04375.x
425.	James, W. P. (2002).	Excluded	Study area difference	https://doi.org/10.1046/j.1440-6047.11.supp3.12.x
426.	Janitz, A. E., et al. (2020).	Excluded	Study area difference	https://doi.org/10.1002/bdr2.1631
427.	Jaruratanasirikul, S., et al. (2014).	Excluded	Study area difference	https://doi.org/10.1007/s00381-014-2410-y
428.	Jebena, M. G., et al. (2016)	Excluded	Unrelated topic	https://doi.org/10.1371/journal.pone.0165931
429.	Jentink, J., et al. (2008).	Excluded	Study area difference	https://doi.org/10.1093/eurpub/ckm129
430.	Jia, S., et al. (2019).	Excluded	Study area difference	https://doi.org/10.1016/j.ijdevneu.2019.09.006
431.	Jin, L., et al. (2017)	Excluded	Study area difference	https://doi.org/10.1111/ppe.12354
432.	Johnson, M. P., et al. (2016)	Excluded	Unrelated topic	https://doi.org/10.1016/j.ajog.2016.07.052

433.	Johnson, M. P., et al. (2016).	Excluded	Study area difference	https://doi.org/10.1016/j.ajog.2016.07.052
434.	Jongbloet, P. H., et al. (2008).	Excluded	Study area difference	https://doi.org/10.1186/1743-1050-5-5
435.	Joray, M. L., et al. (2015)	Excluded	Unrelated topic	https://doi.org/10.1016/j.nutres.2014.10.006
436.	Joubert, B. R., et al. (2016).	Excluded	Unrelated topic	https://doi.org/10.1038/ncomms10577
437.	Julvez, J., et al. (2009)	Excluded	Unrelated topic	https://doi.org/10.1111/j.1365-3016.2009.01032.x
438.	Kahn, L., et al. (2014)	Excluded	Study area difference	https://pubmed.ncbi.nlm.nih.gov/24688343/
439.	Kakebeen, A. D. and L. Niswander (2021).	Excluded	Study area difference	https://doi.org/10.1002/dvg.23455
440.	Källén, B., et al. (2010).	Excluded	Study area difference	https://doi.org/10.1002/bdra.20645
441.	Kalra, S., et al. (2016).	Excluded	Study area difference	https://doi.org/10.1016/j.reprotox.2016.09.005
442.	Kalucy, M., et al. (1994).	Excluded	Study area difference	https://doi.org/10.1111/j.1365-3016.1994.tb00467.x
443.	Kancherla, V. (2023).	Excluded	Study area difference	https://doi.org/10.1007/s00381-023-05910-7
444.	Kant, S., et al. (2017)	Excluded	Study area difference	https://doi.org/10.1002/bdra.23578
445.	Kaplan, E. H., et al. (1998)	Excluded	Unrelated topic	https://doi.org/10.1097/00042560-199804150-00012
446.	Kar, A., et al. (2015)	Excluded	Study area difference	https://doi.org/10.4103/0377-4929.162832
447.	Karakoyun, D. O. and Y. Duzenli (2021).	Excluded	Study area difference	https://doi.org/10.5137/1019-5149.jtn.32532-20.2
448.	Kassie, A., et al. (2022)	Excluded	Unrelated topic	https://doi.org/10.1186/s12884-022-04753-5
449.	Kebede, A., et al. (2019)	Excluded	Unrelated topic	https://doi.org/10.1371/journal.pone.0225205
450.	Kebede, Y. B., et al. (2019)	Excluded	Unrelated topic	https://doi.org/10.1186/s12889-019-7675-3
451.	Kebede, Y., et al. (2020)	Excluded	Unrelated topic	https://doi.org/10.1186/s12936-020-03401-7
452.	Keenan, J. D., et al. (2016)	Excluded	Unrelated topic	https://doi.org/10.1093/jpids/piu143
453.	Keenan, J. D., et al. (2019)	Excluded	Unrelated topic	https://doi.org/10.1371/journal.pntd.0007442
454.	Kefale, B. A., et al. (2023).	Excluded	Unrelated topic	https://doi.org/10.1186/s12887-023-04043-9
455.	Keil, C., et al. (2010)	Excluded	Unrelated topic	https://doi.org/10.1155/2010/213960
456.	Kelbessa, Z., et al. (2014)	Excluded	Unrelated topic	https://doi.org/10.1186/1472-6963-14-324
457.	Kelly, M. S., et al. (2020).	Excluded	Study area difference	https://doi.org/10.3233/prm-190667
458.	Kerr, S. M., et al. (2017).	Excluded	Study area difference	https://doi.org/10.1016/j.annepidem.2017.10.010
459.	Kerr, S. M., et al. (2020)	Excluded	Study area difference	https://doi.org/10.1002/bdr2.1789
460.	Khalid, S. I., et al. (2023).	Excluded	Study area difference	https://doi.org/10.1007/s00381-023-05945-w
461.	Khattak, S. T., et al. (2010).	Excluded	Study area difference	https://pubmed.ncbi.nlm.nih.gov/22455263/
462.	Khodr, Z. G., et al. (2014).	Excluded	Study area difference	https://pubmed.ncbi.nlm.nih.gov/24706436/
463.	Khoshnood, B., et al. (2015).	Excluded	Study area difference	https://doi.org/10.1136/bmj.h5949
464.	Khoury, M. J., et al. (1982).	Excluded	Study area difference	https://doi.org/10.1093/oxfordjournals.aje.a113335

465.	Kibret, G. D., et al. (2019)	Excluded	Unrelated topic	https://doi.org/10.1186/s40249-019-0594-9
466.	Kibret, G. D., et al. (2022)	Excluded	Unrelated topic	https://doi.org/10.1136/bmjopen-2021-058648
467.	Kim, J., et al. (2018).	Excluded	Study area difference	https://doi.org/10.1080/19338244.2017.1356259
468.	Kim, S., et al. (2015).	Excluded	Study area difference	https://doi.org/10.1016/j.apmr.2015.02.029
469.	Kinsman, S. L. and M. C. Doehring (1996)	Excluded	Study area difference	https://doi.org/10.1055/s-2008-1071031
470.	Kirby, R. S. (2017)	Excluded	Study area difference	https://doi.org/10.1053/j.semperi.2017.07.004
471.	Kirstein, O. D., et al. (2018)	Excluded	Unrelated topic	https://doi.org/10.1016/j.actatropica.2018.04.005
472.	Kishimba, R. S., et al. (2015).	Excluded	Study area difference	https://doi.org/10.11604/pamj.2015.20.153.4492
473.	Kitisomprayoonkul, N. and T. Tongsong (2001)	Excluded	Study area difference	https://pubmed.ncbi.nlm.nih.gov/11460957/
474.	Klusmann, A., et al. (2005).	Excluded	Study area difference	https://doi.org/10.1080/08035250500340396
475.	Ko, L. K., et al. (2019)	Excluded	Unrelated topic	https://doi.org/10.1016/j.pvr.2018.12.003
476.	Kondo, A., et al. (2013)	Excluded	Study area difference	https://doi.org/10.1002/bdra.23179
477.	Kondo, A., et al. (2015)	Excluded	Study area difference	https://doi.org/10.1017/s0007114515001439
478.	Kondo, A., et al. (2019)	Excluded	Study area difference	https://doi.org/10.1111/cga.12333
479.	Koning, M., et al. (2023)	Excluded	Unrelated topic	https://doi.org/10.1007/s00381-023-05932-1
480.	Kourtis, A. P., et al. (2023)	Excluded	Study area difference	https://doi.org/10.1016/s2352-3018(23)00108-x
481.	Kousa, Y. A., et al. (2019)	Excluded	Study area difference	https://doi.org/10.1093/hmg/ddz010
482.	Kraay, A. N. M., et al. (2022)	Excluded	Unrelated topic	https://doi.org/10.1016/j.vaccine.2022.09.072
483.	Krajewski, A. K., et al. (2021).	Excluded	Study area difference	https://doi.org/10.1002/bdr2.1788
484.	Krzesinski, E. I., et al. (2019).	Excluded	Study area difference	https://doi.org/10.7196/samj.2019.v109i9.13863
485.	Kshettry, V. R., et al. (2014).	Excluded	Study area difference	https://doi.org/10.3171/2014.3.peds13597
486.	Kumar, M., et al. (2020).	Excluded	Study area difference	https://doi.org/10.1007/s13224-020-01311-x
487.	Kurdi, A. M., et al. (2019)	Excluded	Study area difference	https://doi.org/10.1136/bmjopen-2018-026351
488.	Lacy, M., et al. (2018).	Excluded	Study area difference	https://doi.org/10.1159/000488460
489.	Lafond, K. E., et al. (2016)	Excluded	Unrelated topic	https://doi.org/10.1371/journal.pmed.1001977
490.	Laillou, A., et al. (2020)	Excluded	Unrelated topic	https://doi.org/10.3390/nu12123698
491.	Lakew, Y., et al. (2015)	Excluded	Unrelated topic	https://doi.org/10.1136/bmjopen-2015-008669
492.	Langlois, P. H., et al. (2012).	Excluded	Study area difference	https://doi.org/10.1002/bdra.23045
493.	Lauda-Świeciak, A., et al. (2016).	Excluded	Study area difference	https://doi.org/10.5603/gp.2016.0073
494.	Lavery, A. M., et al. (2014).	Excluded	Study area difference	https://doi.org/10.1002/bdra.23236
495.	Leal da Cruz, M., et al. (2017).	Excluded	Study area difference	https://doi.org/10.1016/j.juro.2016.12.014

496.	Lee, H. Y., et al. (2019)	Excluded	Unrelated topic	https://doi.org/10.1080/16549716.2019.1581467
497.	Lei, Y., et al. (2013)	Excluded	Study area difference	https://doi.org/10.1080/16549716.2019.1581467
498.	Leidinger, A., et al. (2018).	Excluded	Study area difference	https://doi.org/10.1016/j.wneu.2018.06.050
499.	Lester, F. T. (1990)	Excluded	Unrelated topic	https://pubmed.ncbi.nlm.nih.gov/2307153/
500.	Leta, G. T., et al. (2018)	Excluded	Unrelated topic	https://pubmed.ncbi.nlm.nih.gov/30199526/
501.	Leta, G. T., et al. (2020)	Excluded	Unrelated topic	https://doi.org/10.1186/s13071-020-04317-6
502.	Li, K., et al. (2012)	Excluded	Study area difference	https://doi.org/10.46234/ccdcw2022.197
503.	Li, M., et al. (2022).	Excluded	Study area difference	https://doi.org/10.46234/ccdcw2022.197
504.	Li, N., et al. (2016).	Excluded	Study area difference	https://doi.org/10.1097/hjh.0000000000001069
505.	Li, X., et al. (2013)	Excluded	Study area difference	https://doi.org/10.1186/1471-2458-13-161
506.	Li, Z. W., et al. (2008)	Excluded	Study area difference	https://doi.org/10.1002/bdra.20743
507.	Li, Z., et al. (2003)	Excluded	Study area difference	https://doi.org/10.1016/s0140-6736(03)12390-2
508.	Li, Z., et al. (2006)	Excluded	Study area difference	https://doi.org/10.18632/oncotarget.19890
509.	Li, Z., et al. (2013).	Excluded	Study area difference	https://doi.org/10.1007/978-981-99-1659-7_64
510.	Liao, Y., et al. (2010)	Excluded	Study area difference	https://doi.org/10.1080/09603123.2010.491854
511.	Lifson, A. R., et al. (2021).	Excluded	Unrelated topic	https://doi.org/10.1080/09540121.2020.1785999
512.	Lima, N. N. R., et al. (2022).	Excluded	Unrelated topic	https://doi.org/10.1016/j.chiabu.2022.105571
513.	Lindstrom, D. P., et al. (2022)	Excluded	Unrelated topic	https://doi.org/10.1186/s12978-021-01239-z
514.	Liu, J., et al. (2018).	Excluded	Study area difference	https://doi.org/10.1186/s12937-018-0421-3
515.	Liu, S., et al. (2019)	Excluded	Study area difference	https://doi.org/10.1111/ppe.12543
516.	Liu, T., et al. (2018).	Excluded	Study area difference	https://doi.org/10.1016/j.juro.2017.11.04 8
517.	Liu, Y., et al. (2019)	Excluded	Study area difference	https://doi.org/10.1016/j.preghy.2019.09.020
518.	Liu, Z., et al. (2012)	Excluded	Study area difference	https://doi.org/10.3969/j.issn.1673-5374.2011.06.010
519.	Liu, Z., et al. (2019)	Excluded	Study area difference	https://doi.org/10.3760/cma.j.issn.0253-9624.2019.04.016
520.	Lo, A., et al. (2014)	Excluded	Study area difference	https://doi.org/10.7189/jogh.04.010402
521.	Lowry, R. B., et al. (2019).	Excluded	Study area difference	https://doi.org/10.1002/bdr2.1438
522.	Lu, W., et al. (2008).	Excluded	Study area difference	https://doi.org/10.1002/bdra.20474
523.	Luben, T. J., et al. (2009)	Excluded	Study area difference	https://doi.org/10.1016/j.healthplace.2009.02.006
524.	Luo, L., et al. (2021)	Excluded	Study area difference	https://doi.org/10.1002/bdr2.1823
525.	Lupo, P. J., et al. (2010)	Excluded	Study area difference	https://doi.org/10.1002/bdra.20671
526.	Lupo, P. J., et al. (2014).	Excluded	Study area difference	https://doi.org/10.1159/000488460
527.	Meijer, W. M., et al. (2005)	Excluded	Unrelated topic	https://doi.org/10.1016/j.reprotox.2005.01.008
528.	Mekonnen, A. and B. Petros (2016).	Excluded	Unrelated topic	https://pubmed.ncbi.nlm.nih.gov/29115116/
529.	Mekonnen, A., et al. (2018)	Excluded	Unrelated topic	https://doi.org/10.5588/ijtld.18.0029
530.	Mekonnen, A., et al. (2020)	Excluded	Unrelated topic	https://doi.org/10.1186/s12889-020-08788-1

531.	Mekonnen, B. D. and C. A. Wubneh (2020).	Excluded	Unrelated topic	https://doi.org/10.1186/s12978-020-01032-4
532.	Mekonnen, D., et al. (2014)	Excluded	Unrelated topic	https://doi.org/10.11604/pamj.2014.17.40.2465
533.	Mekonnen, F. A., et al. (2018)	Excluded	Unrelated topic	https://doi.org/10.1371/journal.pone.0202734
534.	Mekonnen, S. A., et al. (2021).	Excluded	Unrelated topic	https://doi.org/10.1371/journal.pone.0262032
535.	Mekonnen, Z., et al. (2017)	Excluded	Unrelated topic	https://doi.org/10.1371/journal.pone.0184665
536.	Melaku, Y. A., et al. (2014)	Excluded	Unrelated topic	https://doi.org/10.1186/s12884-014-0418-7
537.	Melaku, Z., et al. (2015)	Excluded	Unrelated topic	https://doi.org/10.1186/s12889-015-1776-4
538.	Melese, G., et al. (2020)	Excluded	Unrelated topic	https://doi.org/10.1186/s12905-020-01041-2
539.	Melkie, M., et al. (2012)	Excluded	Unrelated topic	https://doi.org/10.1186/1756-0500-5-493
540.	Melo, J. R., et al. (2015).	Excluded	Study area difference	https://doi.org/10.1590/0004-282x20150110
541.	Memirie, S. T., et al. (2023)	Excluded	Unrelated topic	https://doi.org/10.1371/journal.pmed.1004198
542.	Mengistu, G. T. and B. K. Mengistu (2023).	Excluded	Unrelated topic	https://doi.org/10.1136/bmjopen-2022-063258
543.	Mengistu, M. (1993)	Excluded	Unrelated topic	https://pubmed.ncbi.nlm.nih.gov/8436098/
544.	Merga, M., et al. (2019)	Excluded	Unrelated topic	https://doi.org/10.1177/2150132719896447
545.	Merid, Y., et al. (2021)	Excluded	Unrelated topic	https://doi.org/10.3855/jidc.14742
546.	Mersha, A. M., et al. (2017)	Excluded	Unrelated topic	https://doi.org/10.11604/pamj.supp.2017.27.2.10677
547.	Messinger, C. J., et al. (2020)	Excluded	Unrelated topic	https://doi.org/10.1001/jamapediatrics.2020.3009
548.	Meyer, R. E. and A. M. Siega-Riz (2002)	Excluded	Study area difference	https://pubmed.ncbi.nlm.nih.gov/12353507/
549.	Miao, Q., et al. (2020).	Excluded	Study area difference	https://doi.org/10.3389/fped.2020.573090
550.	Miller, L. C., et al. (2008)	Excluded	Unrelated topic	https://doi.org/10.1007/s10995-007-0274-4
551.	Miller, N. P., et al. (2014)	Excluded	Unrelated topic	https://pubmed.ncbi.nlm.nih.gov/25845072/
552.	Miller, N. P., et al. (2015)	Excluded	Unrelated topic	https://doi.org/10.1371/journal.pone.0142010
553.	Mills, J. L. and C. Signore (2004).	Excluded	Study area difference	https://doi.org/10.1002/bdra.20075
554.	Mills, J. L., et al. (2010)	Excluded	Unrelated topic	https://doi.org/10.3945/ajcn.2009.28865
555.	Mills, J. L., et al. (2010).	Excluded	Study area difference	https://doi.org/10.3945/ajcn.2009.28865
556.	Milunsky, A., et al. (1989).	Excluded	Unrelated topic	https://doi.org/10.1016/0002-9378(89)90501-2
557.	Mirkin, K., et al. (2013).	Excluded	Study area difference	https://doi.org/10.3233/prm-130254
558.	Misganaw, A., et al. (2017)	Excluded	Unrelated topic	https://doi.org/10.1186/s12963-017-0146-0
559.	Misgina, K. H., et al. (2021)	Excluded	Unrelated topic	https://doi.org/10.1136/bmjopen-2020-043484
560.	Mishra, N., et al. (2020)	Excluded	Unrelated topic	https://doi.org/10.1016/j.meegid.2019.104133
561.	Mitchell, L. E. (2008).	Excluded	Study area difference	https://doi.org/10.1002/bdra.20465
562.	Mohamed, M. A. and H. Aly (2012).	Excluded	Study area difference	https://doi.org/10.1038/jp.2011.184

563.	Mohd-Zin, S. W., et al. (2022).	Excluded	Study area difference	https://doi.org/10.3390/genes13060952
564.	Mollalign, H., et al. (2023).	Excluded	Unrelated topic	https://doi.org/10.1371/journal.pone.0284363
565.	Molloy, A. M., et al. (2009)	Excluded	Study area difference	https://doi.org/10.1542/peds.2008-1173
566.	Monteith, S. J., et al. (2005)	Excluded	Study area difference	https://doi.org/10.1016/j.jocn.2004.08.018
567.	Mony, P. K., et al. (2021).	Excluded	Unrelated topic	https://doi.org/10.1136/bmjgh-2021-005905
568.	Moore, C. A., et al. (1997)	Excluded	Study area difference	https://pubmed.ncbi.nlm.nih.gov/9409858/
569.	Morota, N. and S. Ihara (2008)	Excluded	Study area difference	https://doi.org/10.3171/ped/2008/2/9/188
570.	Morris, J. K. and N. J. Wald (2007)	Excluded	Study area difference	https://doi.org/10.1258/096914107781261945
571.	Morris, J. K., et al. (2016)	Excluded	Study area difference	https://doi.org/10.1136/archdischild-2015-309226
572.	Moges N,et al. (2023)	Excluded	Study area difference	https://bmjpaedsopen.bmj.com/content/7/1/e002022
573.	Morris, S. E., et al. (2003).	Excluded	Unrelated topic	https://doi.org/10.1177/003693300304800403
574.	Mosley, B. S., et al. (2007).	Excluded	Study area difference	https://pubmed.ncbi.nlm.nih.gov/17487022/
575.	Mosley, B. S., et al. (2009)	Excluded	Study area difference	https://doi.org/10.1093/aje/kwn331
576.	Mowla, S., et al. (2020)	Excluded	Study area difference	https://doi.org/10.1002/bdr2.1624
577.	Msamati, B. C., et al. (2000)	Excluded	Study area difference	https://doi.org/10.4314/cajm.v46i11.8572
578.	Mulinare, J., et al. (1988).	Excluded	Study area difference	https://pubmed.ncbi.nlm.nih.gov/3184392/
579.	Mulissa, Z., et al. (2020)	Excluded	Unrelated topic	https://doi.org/10.1371/journal.pone.0237703
580.	Muller, F. (2003).	Excluded	Study area difference	https://doi.org/10.1007/s00381-003-0784-3
581.	Mumpe-Mwanja, D., et al. (2019).	Excluded	Study area difference	https://doi.org/10.1186/s12884-019-2542-x
582.	Murshid, W. R., et al. (2000).	Excluded	Study area difference	https://doi.org/10.1159/000028915
583.	Murthy, G. V., et al. (2016)	Excluded	Study area difference	https://doi.org/10.2196/resprot.5722
584.	Mussa, E. C., et al. (2021)	Excluded	Unrelated topic	https://doi.org/10.1186/s12884-019-2542-x
585.	Myers, M. F., et al. (2001)	Excluded	Unrelated topic	https://doi.org/10.1093/aje/154.11.1051
586.	Naeye, R. L. and N. Tafari (1985)	Excluded	Unrelated topic	https://doi.org/10.1111/j.1651-2227.1985.tb10127.x
587.	Nalbandyan, M., et al. (2019)	Excluded	Unrelated topic	https://doi.org/10.1002/ajmg.a.61290
588.	Natsume, N., et al. (2001).	Excluded	Unrelated topic	https://doi.org/10.1067/moe.2001.112948
589.	Neasham, D., et al. (2001).	Excluded	Study area difference	https://doi.org/10.1046/j.1365-3016.2001.0379a.x
590.	Nega, D., et al. (2021)	Excluded	Unrelated topic	https://doi.org/10.1186/s12889-021-12036-5
591.	Negash, W. D., et al. (2023).	Excluded	Unrelated topic	https://doi.org/10.1186/s12889-023-15952-w
592.	Nigatu, D., et al. (2019)	Excluded	Unrelated topic	https://doi.org/10.1046/j.1365-3016.2001.0379a.x
593.	Niguse, S., et al. (2018)	Excluded	Unrelated topic	https://doi.org/10.1186/s13104-018-3410-x
594.	Nilsen, R. M., et al. (2019)	Excluded	Unrelated topic	https://doi.org/10.3390/nu11102300
595.	Northrup, H. and K. A. Volcik (2000).	Excluded	Study area difference	https://doi.org/10.1067/mpp.2000.112052
596.	O'Brien, K. S., et al. (2020)	Excluded	Unrelated topic	https://doi.org/10.4269/ajtmh.19-0695
597.	Obrycki, J. F., et al. (2019).	Excluded	Study area difference	https://doi.org/10.1002/bdr2.1505

598.	Obu, H. A., et al. (2012).	Excluded	Study area difference	https://doi.org/10.1186/1756-0500-5-177
599.	Oliveira, R. T. C., et al. (2023)	Excluded	Study area difference	https://doi.org/10.1007/s00381-022-05779-y
600.	Olney, R. S. and J. Mulinare (2002).	Excluded	Study area difference	https://doi.org/10.1053/sper.2002.34773
601.	Omran, M., et al. (1993)	Excluded	Study area difference	https://doi.org/10.1111/j.1365-3016.1993.tb00427.x
602.	Orr, M., et al. (2002).	Excluded	Study area difference	https://doi.org/10.1078/1438-4639-00126
603.	Osman, K. A., et al. (2020)	Excluded	Unrelated topic	https://doi.org/10.1111/mcn.12955
604.	Oumer, M., et al. (2021)	Excluded	Design difference	https://doi.org/10.1038/s41598-021-02966-w
605.	Oumer, M., et al. (2021).	Excluded	Design difference	https://doi.org/10.1186/s12887-021-02653-9
606.	Ouyang, L., et al. (2015).	Excluded	Study area difference	https://doi.org/10.1016/j.juro.2014.08.092
607.	Owen, T. J., et al. (2000)	Excluded	Study area difference	https://doi.org/10.1111/j.1467-842x.2000.tb00521.x
608.	Pace, N. D., et al. (2019).	Excluded	Study area difference	https://doi.org/10.1002/bdr2.1552
609.	Padula, A. M., et al. (2015).	Excluded	Study area difference	https://doi.org/10.1111/ppe.12244
610.	Palmsten, K., et al. (2022).	Excluded	Study area difference	https://doi.org/10.1002/pds.5435
611.	Pardthaisong, T., et al. (1988)	Excluded	Study area difference	https://doi.org/10.1002/tera.1420380108
612.	Park, C. H., et al. (1992).	Excluded	Study area difference	https://doi.org/10.1093/oxfordjournals.aje.a116470
613.	Parks, S. E., et al. (2011)	Excluded	Study area difference	https://doi.org/10.1002/bdra.20772
614.	Passarelli, S., et al. (2022)	Excluded	Unrelated topic	https://doi.org/10.1093/jn/nxac015
615.	Patel, N. H., et al. (2020).	Excluded	Unrelated topic	https://doi.org/10.1371/journal.pone.0242577
616.	Perry, M. F., et al. (2019)	Excluded	Study area difference	https://doi.org/10.1016/j.ajog.2019.02.029
617.	Persaud, D., et al. (2011)	Excluded	Unrelated topic	https://doi.org/10.1089/aid.2010.0346
618.	Persson, E. K., et al. (2006).	Excluded	Study area difference	https://doi.org/10.1055/s-2007-964868
619.	Persson, E. K., et al. (2007).	Excluded	Study area difference	https://doi.org/10.1007/s00381-007-0324-7
620.	Perveen, F. and S. Tyyab (2007)	Excluded	Study area difference	https://pubmed.ncbi.nlm.nih.gov/17623582/
621.	Petersen, A. B., et al. (2016)	Excluded	Unrelated topic	https://doi.org/10.1186/s12889-016-3588-6
622.	Petersen, A. B., et al. (2018)	Excluded	Unrelated topic	https://doi.org/10.1186/s12905-018-0640-y
623.	Petrova, J. G. and A. Vaktskjold (2009). .	Excluded	Study area difference	https://doi.org/10.1080/00016340902898008
624.	Piro, E., et al. (2020)	Excluded	Study area difference	https://doi.org/10.1186/s13052-020-00836-1
625.	Polfuss, M., et al. (2022).	Excluded	Study area difference	https://doi.org/10.1542/peds.2022-057007
626.	Porco, T. C., et al. (2009)	Excluded	Unrelated topic	https://doi.org/10.1001/jama.2009.1266
627.	Proos, L. A., et al. (2011)	Excluded	Study area difference	https://doi.org/10.1111/j.1651-2227.2011.02335.x
628.	Punchak, M., et al. (2023).	Excluded	Study area difference	https://doi.org/10.3171/2023.4.peds2327
629.	Qazi, G. (2010).	Excluded	Study area difference	https://pubmed.ncbi.nlm.nih.gov/22455258/
630.	Radcliff, E., et al. (2012).	Excluded	Study area difference	https://doi.org/10.1002/bdra.23084
631.	Radcliff, E., et al. (2016).	Excluded	Study area difference	https://doi.org/10.1007/s00381-016-3105-3

632.	Rango, T., et al. (2017)	Excluded	Unrelated topic	https://doi.org/10.1016/j.scitotenv.2017.04.021
633.	Rashid, A., et al. (2023)	Excluded	Unrelated topic	https://doi.org/10.3389/fcimb.2023.1284815
634.	Ratan, S. K., et al. (2008).	Excluded	Study area difference	https://doi.org/10.1007/s00383-008-2167-z
635.	Ray, J. G., et al. (2005)..	Excluded	Study area difference	https://doi.org/10.1097/01.aog.0000151988.84346.3e
636.	Ray, K. J., et al. (2007)	Excluded	Unrelated topic	https://doi.org/10.1186/1471-2334-7-91
637.	Ray, K. J., et al. (2009)	Excluded	Unrelated topic	https://doi.org/10.1371/journal.pntd.0000458
638.	Razzaghi, H., et al. (2016)	Excluded	Study area difference	https://doi.org/10.1007/s10995-016-1948-6
639.	Rebollo, M. P., et al. (2015)	Excluded	Unrelated topic	https://doi.org/10.1007/s00383-008-2167-z
640.	Reefhuis, J. and M. A. Honein (2004).	Excluded	Study area difference	https://doi.org/10.1002/bdra.20065
641.	Reithinger, R., et al. (2013)	Excluded	Unrelated topic	https://doi.org/10.1093/trstmh/trt096
642.	Rios-Blancas, M. J., et al. (2023)	Excluded	Unrelated topic	https://doi.org/10.3389/fpubh.2023.1189861
643.	Rittler, M., et al. (2004)	Excluded	Study area difference	https://doi.org/10.1002/bdra.10131
644.	Rittler, M., et al. (2008).	Excluded	Study area difference	https://doi.org/10.1597/06-250.1
645.	Rosenberg, K. and H. P. McEwan (1991).	Excluded	Study area difference	https://doi.org/10.1177/003693309103600604
646.	Rosenthal, N. A., et al. (2015)	Excluded	Study area difference	https://doi.org/10.1111/ppe.12175
647.	Rosenthal, T., et al. (1990)	Excluded	Unrelated topic	https://pubmed.ncbi.nlm.nih.gov/2258889/
648.	Rosenthal, Y. S., et al. (2019).	Excluded	Study area difference	https://doi.org/10.1016/j.jpedsurg.2018.05.005
649.	Ross, S. and R. L. Naeye (1981)	Excluded	Unrelated topic	https://pubmed.ncbi.nlm.nih.gov/7322714/
650.	Routh, J. C., et al. (2018).	Excluded	Study area difference	https://doi.org/10.1016/j.juro.2017.08.084
651.	Roza, S. J., et al. (2010).	Excluded	Study area difference	https://doi.org/10.1017/s0007114509991954
652.	Rozendaal, A. M., et al. (2013).	Excluded	Study area difference	https://doi.org/10.1007/s10654-013-9849-0
653.	Ryznychuk, M. O., et al. (2018)	Excluded	Study area difference	https://pubmed.ncbi.nlm.nih.gov/29786583/
654.	Sahlu, I., et al. (2014)	Excluded	Unrelated topic	https://doi.org/10.1016/j.jegh.2014.01.001
655.	Salemi, J. L., et al. (2012)	Excluded	Study area difference	https://doi.org/10.1177/003335491212700407
656.	Salih, M. A., et al. (2014)	Excluded	Study area difference	https://pubmed.ncbi.nlm.nih.gov/25551113/
657.	Schwartz, A. D., et al. (2021)	Excluded	Unrelated topic	https://doi.org/10.1007/s10439-021-02800-4
658.	Senbeto Wolde, T., et al. (2023)	Excluded	Unrelated topic	https://doi.org/10.1016/j.srhc.2023.100913
659.	Seyoum, A., et al. (2002)	Excluded	Unrelated topic	https://doi.org/10.4314/eamj.v79i9.9121
660.	Sharma, V., et al. (2020)	Excluded	Unrelated topic	https://doi.org/10.1371/journal.pmed.1003274
661.	Shaw, G. M., et al. (1995).	Excluded	Study area difference	https://doi.org/10.1097/00001648-199505000-00005
662.	Shaw, G. M., et al. (2000)	Excluded	Study area difference	https://doi.org/10.1002/(sici)1096-9926(200005)61:5%3C376::aid-tera9%3E3.0.co;2-j
663.	Shaw, G. M., et al. (2008).	Excluded	Study area difference	https://doi.org/10.1111/j.1365-3016.2008.00964.x

664.	Shibeshi, W., et al. (2019)	Excluded	Unrelated topic	https://doi.org/10.1186/s40360-019-0313-y
665.	Shiferaw, S., et al. (2017)	Excluded	Unrelated topic	https://doi.org/10.1371/journal.pone.0187311
666.	Sime, H., et al. (2018)	Excluded	Unrelated topic	https://doi.org/10.1371/journal.pntd.0006325
667.	Sims-Williams, H. J., et al. (2017)	Excluded	Study area difference	https://doi.org/10.3171/2016.7.peds16296
668.	Sinshaw, W., et al. (2019)	Excluded	Unrelated topic	https://doi.org/10.1186/s12879-019-4241-7
669.	Sohnesen, T. P., et al. (2017)	Excluded	Unrelated topic	https://doi.org/10.1371/journal.pone.0175445
670.	Solomon, E. T., et al. (2021)	Excluded	Unrelated topic	https://doi.org/10.11604/pamj.2021.40.239.29785
671.	Stark, L., et al. (2017)	Excluded	Unrelated topic	https://doi.org/10.1371/journal.pone.0174741
672.	Strassburg, M. A., et al. (1983).	Excluded	Study area difference	https://doi.org/10.1111/j.1469-8749.1983.tb13823.x
673.	Sully, E., et al. (2018)	Excluded	Unrelated topic	https://doi.org/10.1016/j.jadohealth.2017.12.015
674.	Sunderland, R. and J. L. Emery (1979).	Excluded	Study area difference	https://pubmed.ncbi.nlm.nih.gov/95238/
675.	Suphapeetiporn, K., et al. (2008)	Excluded	Study area difference	https://doi.org/10.1016/j.ejpn.2007.07.005
676.	Sutton, M., et al. (2008)	Excluded	Study area difference	https://doi.org/10.1002/bdra.20498
677.	Sweed, Y. and P. Puri (1993).	Excluded	Study area difference	https://doi.org/10.1136/adc.69.1_spec_no.68
678.	Tadele, H., et al. (2023)	Excluded	Unrelated topic	https://doi.org/10.1111/apa.16812
679.	Tadesse, B., et al. (2017)	Excluded	Unrelated topic	https://doi.org/10.1371/journal.pntd.0006080
680.	Tadesse, F., et al. (2016)	Excluded	Unrelated topic	https://doi.org/10.1186/s12889-016-2773-y
681.	Tamene, A., et al. (2023)	Excluded	Unrelated topic	https://doi.org/10.1371/journal.pntd.0006080
682.	Tamirat, K. S., et al. (2020)	Excluded	Unrelated topic	https://doi.org/10.1186/s12913-020-05485-y
683.	Tamire, M., et al. (2022)	Excluded	Unrelated topic	https://doi.org/10.1371/journal.pone.0277348
684.	Tefera, F., et al. (2019)	Excluded	Unrelated topic	https://doi.org/10.1186/s12913-019-4074-5
685.	Tefera, M., et al. (2022)	Excluded	Unrelated topic	https://doi.org/10.1136/bmjopen-2021-055250
686.	Tefera, Z., et al. (2020)	Excluded	Unrelated topic	https://doi.org/10.1186/s12879-020-05080-w
687.	Teferi, W., et al. (2022)	Excluded	Unrelated topic	https://doi.org/10.1186/s12879-022-07460-w
688.	Teklehaimanot, H. D., et al. (2016)	Excluded	Unrelated topic	https://doi.org/10.1186/s12889-016-2918-z
689.	Teklu, A. M., et al. (2017)	Excluded	Unrelated topic	https://doi.org/10.4314/ejhs.v27i1.2s
690.	Teklu, A. M., et al. (2020)	Excluded	Unrelated topic	https://doi.org/10.1186/s12887-020-02311-6
691.	Tesfay, W., et al. (2020)	Excluded	Unrelated topic	https://doi.org/10.1371/journal.pone.0238311
692.	Teshome, S., et al. (2023)	Excluded	Unrelated topic	https://doi.org/10.3390/ijms241813891
693.	Tessema, G. A., et al. (2018)	Excluded	Unrelated topic	https://doi.org/10.1186/s12884-018-1790-5
694.	Tessema, M., et al. (2021)	Excluded	Unrelated topic	https://doi.org/10.1017/s1368980021000422
695.	Tessema, T., et al. (2022)	Excluded	Unrelated topic	https://doi.org/10.1371/journal.pone.0267868
696.	Tessema, Z. T. and S. A. Tiruneh (2020).	Excluded	Unrelated topic	https://doi.org/10.1186/s12884-020-02986-w

697.	Tewodros, W. and G. Kronvall (2005)	Excluded	Unrelated topic	https://doi.org/10.1128/jcm.43.9.4369-4376.2005
698.	Tewodros, W., et al. (1992)	Excluded	Unrelated topic	https://doi.org/10.1017/s0950268800050172
699.	Thong, P. L., et al. (2019).	Excluded	Study area difference	https://pubmed.ncbi.nlm.nih.gov/31424034/
700.	Tibebu, N. S., et al. (2022)	Excluded	Unrelated topic	https://doi.org/10.1186/s12887-022-03253-x
701.	Tinker, S. C., et al. (2012).	Excluded	Study area difference	https://doi.org/10.1002/bdra.23024
702.	Tiruneh, S. A., et al. (2021)	Excluded	Unrelated topic	https://doi.org/10.1371/journal.pone.0252639
703.	Titiyos, A., et al. (2023).	Excluded	Unrelated topic	https://doi.org/10.1186/s12978-023-01709-6
704.	Tola, H. H., et al. (2016)	Excluded	Unrelated topic	https://doi.org/10.1371/journal.pone.0155147
705.	Travassos, M. A., et al. (2016)	Excluded	Unrelated topic	https://doi.org/10.1371/journal.pone.0149970
706.	Tsegaye, B. and W. Ergete (2003).	Excluded	Unrelated topic	https://doi.org/10.4314/eamj.v80i10.8755
707.	Tuji, A., et al. (2023)	Excluded	Unrelated topic	https://doi.org/10.1186/s12888-023-04891-w
708.	Tymejczyk, O., et al. (2016)	Excluded	Unrelated topic	https://doi.org/10.1007/s10461-015-1184-x
709.	Ugwu, R. O., et al. (2007)	Excluded	Study area difference	https://doi.org/10.4314/njm.v16i4.37340
710.	Usman, A. K., et al. (2019)	Excluded	Unrelated topic	https://doi.org/10.1186/s12913-019-4672-2
711.	Vivas, A. P., et al. (2010)	Excluded	Unrelated topic	https://pubmed.ncbi.nlm.nih.gov/21155409/
712.	Volcik, K. A., et al. (2003)	Excluded	Study area difference	https://doi.org/10.1002/bdra.10008
713.	Walelign, S., et al. (2024)	Excluded	Unrelated topic	https://doi.org/10.1002/iid3.1222
714.	Waller, D. K., et al. (2010)	Excluded	Study area difference	https://doi.org/10.1097/ede.0b013e3181c9fbb3
715.	Walsh, S., et al. (2020)	Excluded	Unrelated topic	https://doi.org/10.1038/s41598-020-78081-z
716.	Wang, H., et al. (2016).	Excluded	Study area difference	https://doi.org/10.3390/nu8030152
717.	Wang, M., et al. (2015)	Excluded	Study area difference	https://doi.org/10.3390/nu7053067
718.	Wassie, G. T., et al. (2021)	Excluded	Unrelated topic	https://doi.org/10.1371/journal.pone.0258468
719.	Watanabe, E., et al. (2014)	Excluded	Unrelated topic	https://doi.org/10.1016/j.jegh.2014.06.001
720.	Weldearegawi, B., et al. (2014)	Excluded	Unrelated topic	https://doi.org/10.1371/journal.pone.0093099
721.	White, F. M., et al. (1988).	Excluded	Study area difference	https://pubmed.ncbi.nlm.nih.gov/3275483/
722.	Whiteman, D., et al. (2000)	Excluded	Study area difference	https://doi.org/10.1093/aje/152.9.823
723.	Wilhelm, A. K., et al. (2016)	Excluded	Unrelated topic	https://doi.org/10.1007/s10900-016-0155-0
724.	Wilkes, J. K., et al. (2021)	Excluded	Study area difference	https://doi.org/10.1007/s00246-021-02576-3
725.	Windham, G. C. and L. E. Sever (1982).	Excluded	Study area difference	https://pubmed.ncbi.nlm.nih.gov/7180853/
726.	Wittberg, D. M., et al. (2021)	Excluded	Unrelated topic	https://doi.org/10.1136/bmjopen-2020-039529
727.	Wogayehu, B., et al. (2019)	Excluded	Unrelated topic	https://doi.org/10.1371/journal.pone.0223523
728.	Wondale, B., et al. (2020)	Excluded	Unrelated topic	https://doi.org/10.1186/s12879-020-05394-9
729.	Wong, L. Y. and L. J. Paulozzi (2001).	Excluded	Study area difference	https://doi.org/10.1046/j.1365-3016.2001.00371.x

730.	Woyessa, A. B., et al. (2018)	Excluded	Unrelated topic	https://doi.org/10.1186/s12879-018-3365-5
731.	Woyessa, A. B., et al. (2019)	Excluded	Unrelated topic	https://doi.org/10.11604/pamj.2019.32.202.18188
732.	Wu, J., et al. (2004).	Excluded	Study area difference	https://doi.org/10.1186/1471-2458-4-23
733.	Wu, Y. W., et al. (2006)	Excluded	Study area difference	https://doi.org/10.1002/bdra.20313
734.	Yalew, M., et al. (2023)	Excluded	Unrelated topic	https://doi.org/10.1186/s12884-023-05593-7
735.	Yang, W., et al. (2019).	Excluded	Study area difference	https://doi.org/10.1002/bdr2.1556
736.	Yang, W., et al. (2021)	Excluded	Study area difference	https://doi.org/10.1080/14767058.2019.1647529
737.	Yang, Y., et al. (2023)	Excluded	Study area difference	https://doi.org/10.1007/s11356-022-21962-9
738.	Yavnai, N., et al. (2011)	Excluded	Unrelated topic	https://pubmed.ncbi.nlm.nih.gov/22686105/
739.	Yazdy, M. M., et al. (2012).	Excluded	Study area difference	https://doi.org/10.1002/bdra.23025
740.	Yerby, M. S. (2003).	Excluded	Study area difference	https://doi.org/10.1212/wnl.61.6_suppl_2.s23
741.	Yi, D., et al. (2015).	Excluded	Study area difference	https://doi.org/10.1016/j.neuro.2014.12.003
742.	Yigezu, A., et al. (2023)	Excluded	Unrelated topic	https://doi.org/10.1136/bmjopen-2022-068498
743.	Yitbarek, K., et al. (2019)	Excluded	Unrelated topic	https://doi.org/10.1371/journal.pone.0216962
744.	Yitbarek, K., et al. (2021)	Excluded	Unrelated topic	https://doi.org/10.1016/j.vaccine.2021.06.015
745.	Yu, M., et al. (2015).	Excluded	Study area difference	https://doi.org/10.4103/0366-6999.147785
746.	Zash, R., et al. (2019).	Excluded	Study area difference	https://doi.org/10.1056/nejmoa1905230
747.	Zepro, N. B., et al. (2023)	Excluded	Unrelated topic	https://doi.org/10.1186/s12913-023-09616-z
748.	Zewdie, Z., et al. (2020)	Excluded	Unrelated topic	https://doi.org/10.1186/s12884-020-2838-x
749.	Zhang, B. Y., et al. (2008)	Excluded	Study area difference	https://doi.org/10.1016/s0895-3988(08)60005-7
750.	Zhang, L., et al. (2021)	Excluded	Study area difference	https://doi.org/10.1080/14767058.2019.1606192
751.	Zhang, X., et al. (2012)	Excluded	Study area difference	https://doi.org/10.1186/1471-2431-12-125
752.	Zhao, W., et al. (2006).	Excluded	Study area difference	https://doi.org/10.1002/bdra.20240
753.	Zhu, H., et al. (2004).	Excluded	Study area difference	https://doi.org/10.1016/j.ymgme.2003.11.003
